# Identification of candidate miRNA biomarkers for facioscapulohumeral muscular dystrophy using DUX4-based mouse models

**DOI:** 10.1242/dmm.049016

**Published:** 2021-08-24

**Authors:** Andreia M. Nunes, Monique Ramirez, Takako I. Jones, Peter L. Jones

**Affiliations:** Department of Pharmacology, University of Nevada, Reno School of Medicine, Reno, NV 89557, USA

**Keywords:** DUX4, FSHD, Biomarker, miR-206, miRNA

## Abstract

Facioscapulohumeral muscular dystrophy (FSHD) is caused by misexpression of *DUX4* in skeletal myocytes. As *DUX4* is the key therapeutic target in FSHD, surrogate biomarkers of *DUX4* expression in skeletal muscle are critically needed for clinical trials. Although no natural animal models of FSHD exist, transgenic mice with inducible DUX4 expression in skeletal muscles rapidly develop myopathic phenotypes consistent with FSHD. Here, we established a new, more-accurate FSHD-like mouse model based on chronic DUX4 expression in a small fraction of skeletal myonuclei that develops pathology mimicking key aspects of FSHD across its lifespan. Utilizing this new aged mouse model and DUX4-inducible mouse models, we characterized the DUX4-related microRNA signatures in skeletal muscles, which represent potential biomarkers for FSHD. We found increased expression of *miR-31-5p* and *miR-206* in muscles expressing different levels of DUX4 and displaying varying degrees of pathology. Importantly, *miR-206* expression is significantly increased in serum samples from FSHD patients compared with healthy controls. Our data support *miR-31-5p* and *miR-206* as new potential regulators of muscle pathology and *miR-206* as a potential circulating biomarker for FSHD.

This article has an associated First Person interview with the first author of the paper.

## Abstract (precis)

**Summary:** Candidate miRNA biomarkers for facioscapulohumeral muscular dystrophy (FSHD) were identified using FSHD-like mouse models that present cumulative pathology from chronic expression of DUX4 in skeletal muscles and confirmed in FSHD patient serum.

## INTRODUCTION

Facioscapulohumeral muscular dystrophy (FSHD) is a progressive myopathy that affects ∼1 in 8300-15,000 people worldwide (Deenen et al., 2014; Orphanet, 2021; Padberg et al., 1995). FSHD affects women and men of all ages; however, it is characterized by high variability in clinical presentation with disease onset, progression and severity differing greatly between individuals and particularly within families (Jones et al., 2012; Mul et al., 2018b; Statland et al., 2015a; Tawil et al., 1996; Tonini et al., 2004; Wang and Tawil, 2016). FSHD-specific clinical trials have recently begun, but there is still no treatment or cure. Importantly, these clinical trials have brought to the forefront that one of the greatest unmet needs in the search for an FSHD therapeutic is a validated circulating surrogate biomarker for pathogenic gene expression or disease progression that can be used to readily monitor clinical efficacy of a treatment.

The root cause of FSHD is misexpression of the double homeobox 4 (*DUX4*) retrogene in skeletal muscle (Gabriëls et al., 1999; Kowaljow et al., 2007; Lemmers et al., 2010; Snider et al., 2010; Tassin et al., 2013). Residing within the chromosome 4q35 D4Z4 macrosatellite repeat array, *DUX4* encodes a critical transcription factor that drives the early stages of normal embryonic development (De Iaco et al., 2017; Gabriëls et al., 1999; Hendrickson et al., 2017; Kowaljow et al., 2007; Rickard et al., 2015; Snider et al., 2010). In contrast to its embryonic role, *DUX4* is under strong epigenetic silencing in most adult somatic cells and paradoxically pathogenic when expressed in these cells (Gabriëls et al., 1999; Snider et al., 2010). In all forms of FSHD, expression of the pathogenic *DUX4* mRNA full-length isoform (*DUX4-fl*) is induced upon epigenetic de-repression of the 4q35 D4Z4 locus brought about by genetic mutations (de Greef et al., 2009; Jones et al., 2015; Snider et al., 2010; van Overveld et al., 2003). This epigenetic dysregulation can be induced either by contraction of the D4Z4 array (FSHD1) (van Deutekom et al., 1993; van Overveld et al., 2003; Wijmenga et al., 1992) or by mutations in genes encoding epigenetic repressors of the D4Z4 array (FSHD2) (Hamanaka et al., 2020; Lemmers et al., 2012; van den Boogaard et al., 2016). Thus, the aberrant expression of *DUX4* is the prime target for FSHD therapeutic development.

A major obstacle facing FSHD clinical trials targeting *DUX4* expression is the lack of any independently validated circulating molecules that can reproducibly be used as predictive, prognostic and/or pharmacodynamic biomarkers (Banerji, 2020; Harafuji et al., 2013; Heier et al., 2020; Matsuzaka et al., 2014; Petek et al., 2016; Signoelli et al., 2020; Statland et al., 2014). Circulating biomarkers are ideal candidates as these are non-invasive, typically inexpensive to assess and allow multiple points of analysis over time, compared with biomarkers that must be detected using muscle biopsies or magnetic resonance imaging (MRI). Three previous studies identified three distinctly different candidate protein biomarkers in serum: creatine kinase MB isoform (CKMB), epidermal growth factor and the inflammatory regulatory factor S100A8 (Heier et al., 2020; Petek et al., 2016; Statland et al., 2014). As an alternative to circulating protein biomarkers, a global gene expression analysis was performed using blood from two well-controlled patient cohorts, but failed to find gene expression profiles differentially expressed in FSHD patients (Signoelli et al., 2020). Additional non-invasive approaches such as MRI and ultrasound techniques have been developed as imaging biomarkers of disease progression, and display the sensitivity to define disease severity and motor function (Mul et al., 2018a, 2017; Wang et al., 2019, 2021b). Importantly MRI measurements have shown a correlation with both pathological changes and expression of DUX4 target genes (Wang et al., 2019), but not with immunosuppressive treatments for FSHD (Wang et al., 2021a). However, using MRI to track drug efficacy in a large clinical trial could be severely limiting due to the cost, limited accessibility and inconsistency of equipment across trial sites. Thus, a DUX4-responsive circulating biomarker is still needed in FSHD clinical trials for drugs targeting *DUX4* expression.

Recently, microRNAs (miRNAs) have gained considerable attention as potential circulating disease biomarkers (Dai et al., 2019; Huang, 2017; Leidinger et al., 2013; Zhao et al., 2019). miRNAs are a class of highly conserved small non-coding RNAs that function in the post-transcriptional regulation of gene expression and are important for the healthy development of many organs, including skeletal muscle (Dai et al., 2019; Huang, 2017; Leidinger et al., 2013; Zhao et al., 2019). They function to tune gene expression by binding to target mRNAs and either inducing their degradation or inhibiting their translation (Galagali and Kim, 2020). Importantly, miRNAs can be transported extracellularly and are found in serum, where their levels may indicate specific disease states (Dai et al., 2019; Huang, 2017; Leidinger et al., 2013; Zhao et al., 2019). For example, microRNA-206 (*miR-206*), one of the original myomiRs, or muscle-specific miRNAs, is a key regulator of myogenic differentiation during muscle repair (Cacchiarelli et al., 2011b; Chen et al., 2010; Greco et al., 2009; Horak et al., 2016; Kim et al., 2006; Liu et al., 2012; Ma et al., 2015; Yuasa et al., 2008). Interestingly, *miR-206* levels are elevated in muscle biopsies from the *mdx* mouse model of Duchenne muscular dystrophy (DMD) and in serum and muscle biopsies isolated from DMD patients (Cacchiarelli et al., 2011b; Mizuno et al., 2011; Roberts et al., 2012, 2013). Other miRNAs have been shown to play major roles in skeletal myogenesis and regeneration, further supporting the use of miRNAs as biomarkers for muscle diseases (Buckingham and Rigby, 2014; Liu and Olson, 2010; McCarthy, 2008; Sempere et al., 2004). For example, a large miRNA profile performed using patient muscle biopsies demonstrated that *miR-146b*, *miR-221*, *miR-155*, *miR-214* and *miR-222* are consistently dysregulated in many muscular dystrophies (Eisenberg et al., 2007). With respect to miRNAs in FSHD, characterization of quadriceps muscle biopsies taken from fetal tissue revealed that *miR-34a*, *miR-330*, *miR-331-5p*, *miR-380-3p*, *miR-516b*, *miR-517*, *miR-582-5p* and *miR-625* are exclusively expressed in FSHD1 compared with control fetal tissue (Portilho et al., 2015). In addition, a recent miRNA screen of FSHD patient serum using a panel of 384 miRNAs identified eight as being increased in FSHD patients compared with healthy controls, thus warranting further investigation (Heier et al., 2020). Overall, despite recent advances, no circulating disease biomarkers, including miRNAs, have been validated as being DUX4 responsive for use in FSHD clinical trials.

To address this need, we used our recently developed tamoxifen (TMX)-inducible DUX4 bi-transgenic mouse line, *ACTA1-MCM;FLExD*, that recapitulates many aspects of FSHD pathophysiology of varying severity in skeletal muscles (Jones et al., 2020). Interestingly, in the absence of induction, these mice show chronic mosaic DUX4 expression in all skeletal muscles over their lifetime, developing FSHD-like pathophysiology that accumulates with age, thereby representing a new FSHD-like model of chronic DUX4 expression. Here, we used this model as a discovery tool for identifying the DUX4-reponsive miRNA signatures associated with short-term and chronic exposure to DUX4 in skeletal muscles and demonstrated that *miR-31-5p* and *miR-206* levels are consistently dysregulated. Importantly, levels of *miR-206* are also increased in serum isolated from FSHD patients, thus validating *miR-206* as a potential circulating DUX4-responsive disease biomarker for FSHD.

## RESULTS

### Immune/regeneration-related muscle miRNA profile in DUX4-induced FSHD-like mouse model

**Fig. 1.**
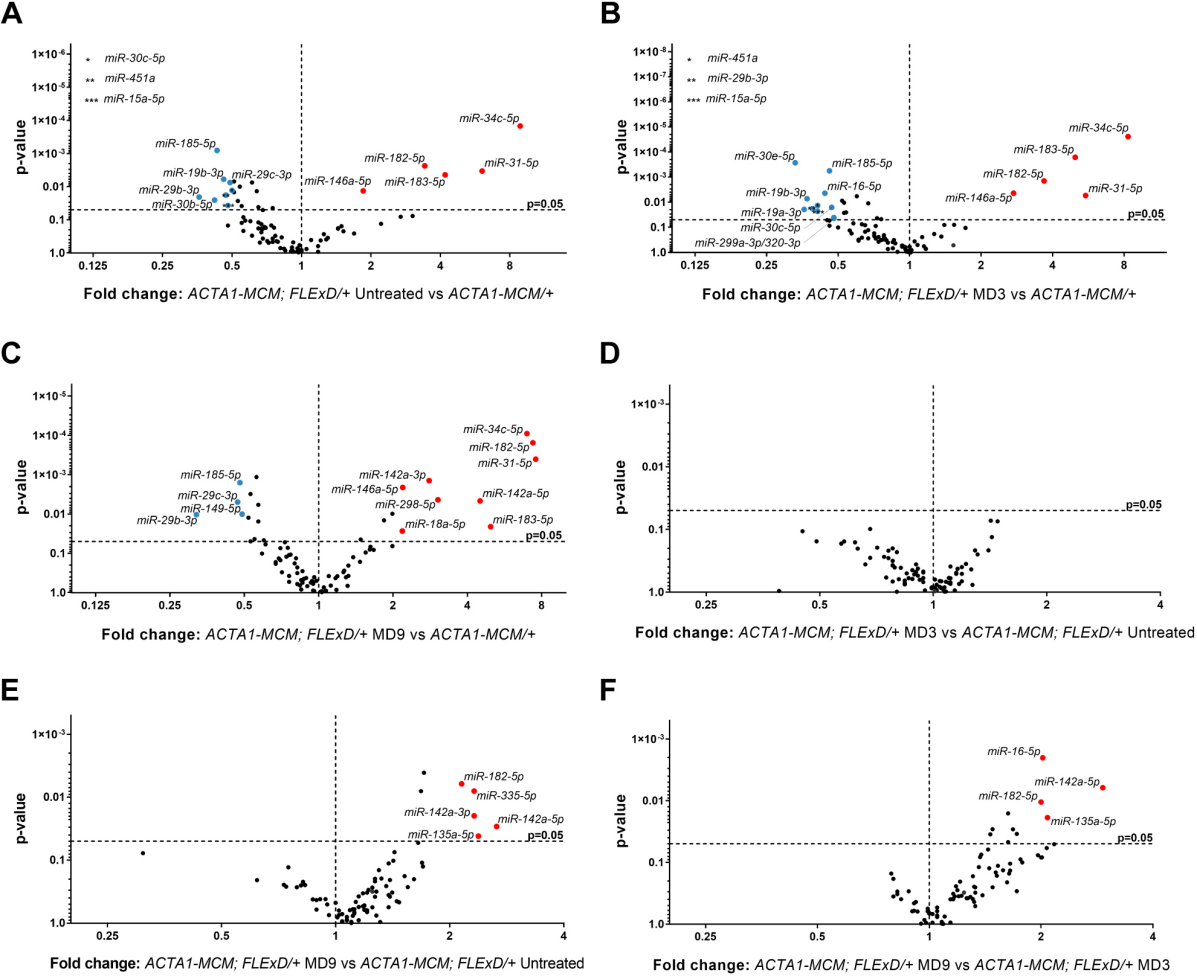
**The miRNA profile in gastrocnemius muscle from DUX4-induced FSHD-like mouse models.** (A-C) Volcano plots depict the miRNA profile in gastrocnemius muscle from FSHD-like models compared with *ACTA1-MCM/+* controls for 3-month-old untreated (no TMX) *ACTA1-MCM;FLExD/+* mice (A), moderate FSHD-like model mice, day 3 (MD3) (B) and moderate FSHD-like model mice, day 9 (MD9) (C). (D,E) Volcano plots for the miRNA profiles for MD3 (D) and MD9 (E) compared with *ACTA1-MCM FLExD/+* untreated mice. (F) The miRNA profile for MD9 compared with MD3 gastrocnemius muscle. Red and blue circles depict miRNAs for which levels are significantly upregulated or downregulated by ≥2-fold, respectively. All studies were performed with *n*=8 animals for each experimental group. Volcano plots represent two-tailed unpaired Student's *t*-test analysis.

We used FSHD-like mouse models as a primary discovery tool to identify miRNAs altered by variable DUX4 expression in skeletal muscles for evaluation as FSHD biomarkers in serum analysis. We first used our laboratory's moderate FSHD-like mouse model (Jones et al., 2020) to evaluate the effect of DUX4 levels on the muscle miRNA signature. As previously published, DUX4 expression is very low in muscles of untreated 3-month-old *ACTA1-MCM;FLExD/+* mice [no TMX in Jones et al. (2020)]. Following TMX induction (single dose, 5 mg/kg), increased DUX4 protein is first detectable by day 3 [moderate day (MD)3], with levels increasing until day 14 (MD14). The increase in DUX4 is accompanied by increased immune cell infiltration, damaged/split myofibers showing variable diameter, centrally localized nuclei and embryonic myosin expression in murine skeletal muscles, all consistent with an FSHD-like phenotype (Jones et al., 2020). Thus, based on this shared histopathology between our FSHD-like mouse and FSHD patients, we chose an immune-related miRNA panel and screened the gastrocnemius muscles from these mice over a time course of DUX4 induction (Fig. 1; Fig. S1, Table S1). Comparing the miRNA profiles from the gastrocnemius muscles, we found that *miR-31-5p*, *miR-34c-5p*, *miR-146a-5p*, *miR-182-5p* and *miR-183-5p* were significantly upregulated by more than 2-fold in the muscles from untreated low-DUX4 mice compared with those of age-matched DUX4-negative control *ACTA1-MCM/+* mice (Fig. 1A). When moderate expression of DUX4 was induced by TMX injection, these miRNAs remained upregulated in MD3 and MD9 muscles compared with control muscles (Fig. 1B,C). In addition, the levels of *miR-18a-5p*, *miR-142a-3p*, *miR-142-5p* and *miR-298-5p* were also upregulated in MD9 muscles (Fig. 1C). To see whether the miRNA profile is sensitive to the progressive increase in DUX4 levels after DUX4 induction, we compared the miRNA profile of *ACTA1-MCM;FLExD/+* MD3 and MD9 muscles with the profile of low DUX4-expressing untreated *ACTA1-MCM;FLExD/+* muscles (Fig. 1D-F). We found no significant miRNA dysregulation at MD3 compared with untreated *ACTA1-MCM;FLExD/+* mice, which suggests that subtle changes in DUX4 expression do not immediately affect certain miRNAs (Fig. 1D). However, as expected, muscles from the *ACTA1-MCM;FLExD/+* MD9 mice expressed higher levels of immune-related *miR-142a-5p* and *miR-182-5p*, as well as *miR-135a-5*, which are associated with muscle regeneration (Fig. 1E,F), consistent with the DUX4-related histopathology (Jones et al., 2020). This indicates that their increases in expression similarly coincide with the increased levels of DUX4 expression, inflammation, regeneration and overall muscle pathology (Fig. 1E,F) (Jones et al., 2020).

**Fig. 2.**
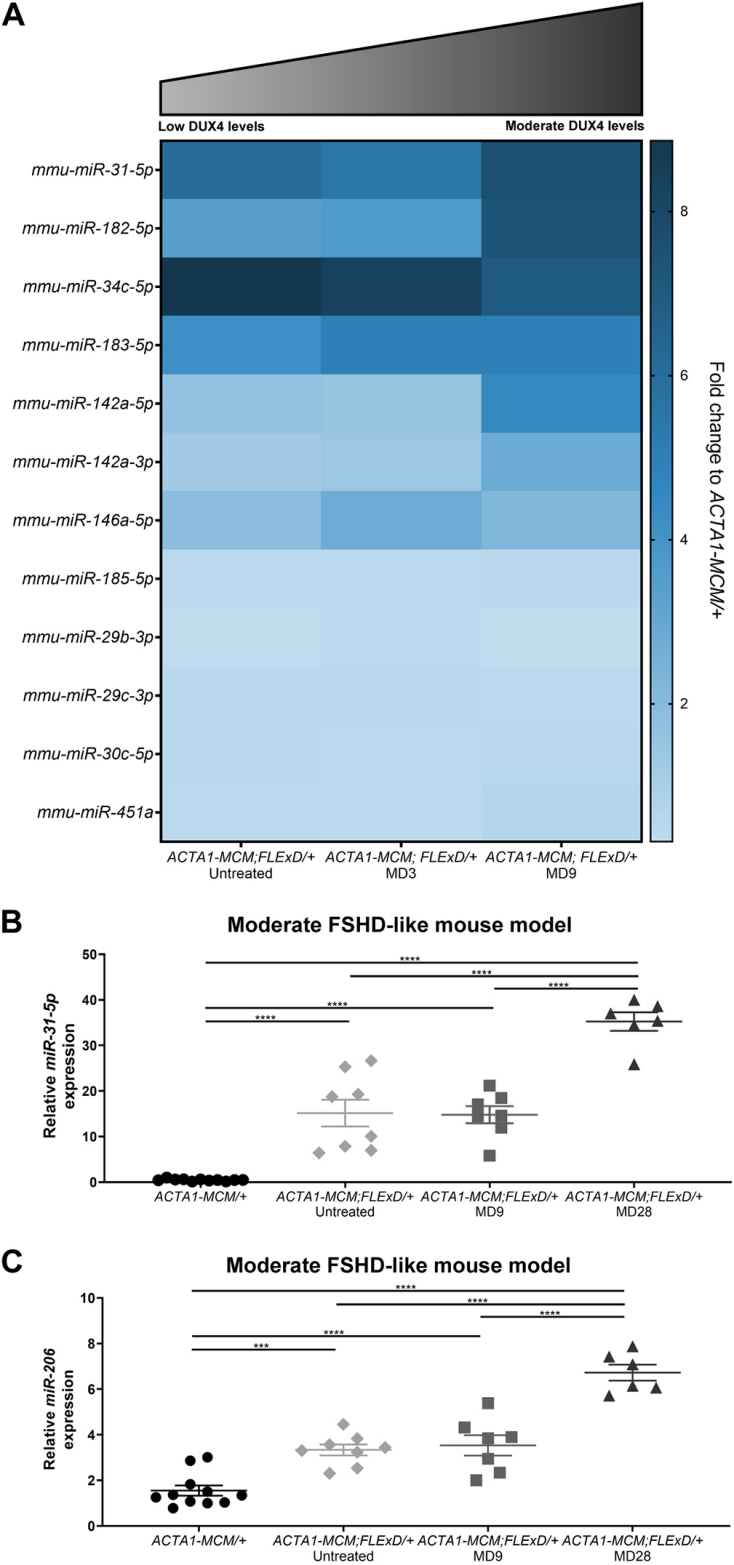
**The miRNA profile dynamics in DUX4-induced FSHD-like mouse models.** (A) Heat map for significantly dysregulated miRNAs in untreated and DUX4-induced *ACTA1-MCM;FLExD/+* models (*n*=8 animals for each experimental group). Schematic depicts DUX4 levels in untreated and DUX4-induced *ACTA1-MCM;FLExD/+* models. (B,C) TaqMan qRT-PCR quantification of *miR-31-5p* (B) and *miR-206* (C) in gastrocnemius muscle from 3-month-old *ACTA1-MCM/+* control mice, untreated (no TMX) *ACTA1-MCM;FLExD/+* mice, moderate FSHD-like model mice, day 9 (MD9) and moderate FSHD-like model mice, day 28 (MD28) (*n*=6-11 for each group). Statistical analysis was performed with two-tailed one-way ANOVA (uncorrected Fisher's LSD) with *n*=11 animals for *ACTA1-MCM/+*, *n*=8 animals for *ACTA1-MCM;FLExD/+* untreated, *n*=7 animals for *ACTA1-MCM;FLExD/+* MD9 and *n*=6 animals for *ACTA1-MCM;FLExD/+* MD28 experimental groups. Data are presented as mean±s.e.m.; ***P<0.001, ****P<0.0001.

The miRNA profile analysis of the moderate FSHD-like mouse model revealed significantly increased expression of multiple miRNAs after DUX4 induction (Fig. 1 and Fig. 2A). Although several of the dysregulated miRNAs did not appear to be sensitive to DUX4 levels, there was a general trend toward increasing levels for seven of the 12 dysregulated miRNAs (e.g. *miR-142a-3p* and *miR-182-5p*), with increasing levels of DUX4 in the muscles (Fig. 2A). The most striking upregulation was found with the immune- and regeneration-related *miR-31-5p*, the levels of which were increased 6-fold in untreated muscles and 8-fold in MD9 muscles (Fig. 2A). By using more-sensitive TaqMan probe quantification, we confirmed the significant upregulation of *miR-31-5p* levels by 15-fold in untreated and MD9 muscles compared with *ACTA1-MCM/+* (DUX4-negative) controls, validating *miR-31-5p* as a potential early biomarker for DUX4 expression levels (Fig. 2B). However, *miR-31-5p* levels continued to increase by 35-fold in the MD28 *ACTA1-MCM;FLExD/+* muscles, which have very low DUX4 expression (Jones et al., 2020). This suggests that although increased levels of *miR-31-5p* are DUX4 dependent, expression of this miRNA is not DUX4 responsive (i.e. does not decrease with decreasing DUX4 levels) and thus may reflect the activation of DUX4-induced pathology.

In addition to the immune/regeneration-related miRNA array, we also analyzed another regeneration-related miRNA, *miR-206*, which has been proposed as a biomarker for DMD and is a key regulator of myogenesis, muscle differentiation and muscle repair (Cacchiarelli et al., 2011b; Mizuno et al., 2011; Roberts et al., 2012, 2013). Interestingly, the TaqMan probe assay revealed that *miR-206* was increased by 3- and 4-fold in untreated and MD9 *ACTA1-MCM;FLExD/+* muscles, respectively, compared with *ACTA1-MCM/+* control, indicating that even low DUX4 expression is sufficient to induce a significant dysregulation of *miR-206* expression (Fig. 2C). As with *miR-31-5p*, levels of *miR-206* were further increased in MD28 *ACTA1-MCM;FLExD/+* muscles (Fig. 2C), similarly suggesting that its expression may correlate with DUX4-induced pathology.

The miRNA profile analysis also identified several miRNAs for which levels were significantly decreased in DUX4-induced muscle (Fig. 1). Expression levels for *miR-15a-5p*, *miR-19b-3p*, *miR-29b-3p*, *miR-185-5p* and *miR-451a* were decreased in both untreated and MD3 *ACTA1-MCM;FLExD/+* muscles (Fig. 1A,B), and *miR-29c-3p* and *miR-149-5p* levels were uniquely downregulated in MD9 *ACTA1-MCM;FLExD/+* muscles (Fig. 1C). However, only *miR-29b-3p* and *miR-185-5p* levels were consistently decreased in all DUX4-expressing muscles (Fig. 1A-C), and therefore they are also potential candidates for FSHD muscle biomarkers. Overall, our results reveal that DUX4 expression results in a dynamic miRNA profile in murine skeletal muscle.

### Chronic DUX4-expressing FSHD-like mouse model

The short-term increase in mosaic bursting of DUX4 expression in skeletal muscles caused by TMX induction in the moderate *ACTA1-MCM;FLExD* FSHD-like mouse model was useful for pushing the system and enabling candidate DUX4-reponsive biomarker discovery; however, it is not very representative of the situation found in FSHD patients, who experience chronic, low levels of mosaic DUX4 expression in skeletal muscles, leading to accumulated muscle damage over their lifetimes. Fortunately, the *ACTA1-MCM;FLExD/+* mouse similarly expresses very low levels of mosaic DUX4 expression in skeletal muscles throughout its lifetime in the absence of TMX induction (Jones and Jones, 2018; Jones et al., 2020). This chronic, skeletal muscle-specific expression of DUX4 is a result of the *ACTA1* [or human skeletal actin promoter (HSA)]-regulated mutated estrogen receptor dimer fused with cre (MCM) protein leaking into a small fraction of myonuclei of *ACTA1-MCM;FLExD* mice. This leads to sporadic transgene recombination in the absence of TMX induction exclusively in skeletal muscles, resulting in a mosaic expression pattern in rare myonuclei, and suggests a potentially better model for recapitulating FSHD (Jones et al., 2020).

**Fig. 3.**
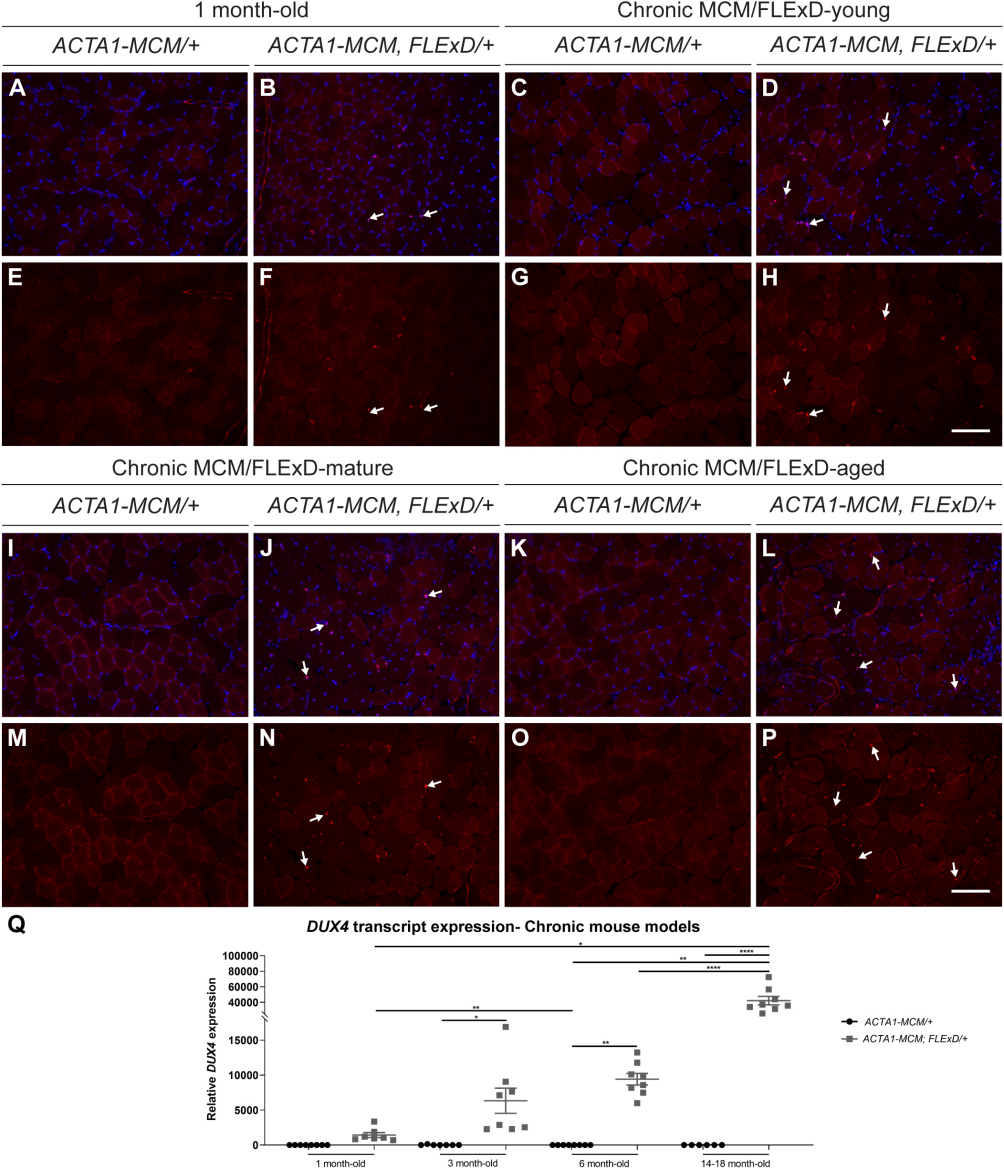
**DUX4 expression in chronic FSHD-like mouse models.** (A-P) Immunofluorescence staining for DUX4 (red) and DAPI (blue) in gastrocnemius muscle isolated from 1-month-old *MCM/FLExD* mice (A,B,E,F), 3-month-old or chronic MCM/FLExD-young mice (C,D,G,H), 6-month-old or chronic MCM/FLExD-mature mice (I,J,M,N) and 14- to 18-month-old or chronic MCM/FLExD-aged mice (K,L,O,P), compared with control *ACTA1-MCM/+* mice. Representative images from *n*=3 animals for each experimental group. Scale bars: 100 µm. (Q) *DUX4* expression in the gastrocnemius muscle isolated from 1 month-old, chronic MCM/FLExD-young, chronic MCM/FLExD-mature and chronic MCM/FLExD-aged mice, compared with age-matched *ACTA1-MCM/+* controls. Statistical analysis was performed with two-tailed one-way ANOVA (uncorrected Fisher's LSD) with *n*=8 animals for *ACTA1-MCM/+* 1-month-old, *n*=7 animals for *ACTA1-MCM;FLExD/+* 1-month-old, *n*=7 animals for *ACTA1-MCM/+* 3-month-old, *n*=8 animals for chronic MCM/FLExD-young, *n*=8 animals for *ACTA1-MCM/+* 6-month-old, *n*=8 animals for chronic MCM/FLExD-mature, *n*=8 animals for *ACTA1-MCM/+* 14- to 18-month-old and *n*=8 animals for chronic MCM/FLExD-aged experimental groups. Data are presented as mean±s.e.m.; *P<0.05, **P<0.01, ****P<0.0001.

We quantified DUX4 expression in the gastrocnemius muscle of 1-, 3-, 6- and 14- to 18-month-old *ACTA1-MCM;FLExD/+* mice and their age-matched *ACTA1-MCM/+* controls using immunohistochemistry and quantitative reverse transcription PCR (qRT-PCR) (Fig. 3). Hereafter, we will refer to 3-month-old *ACTA1-MCM;FLExD/+* mice as chronic MCM;FLExD-young (equivalent to human age 20 years), whereas 6-month-old mice will be referred to as chronic MCM;FLExD-mature (equivalent to human age 30 years) and 14- to 18-month-old mice as chronic MCM;FLExD-aged (equivalent to human age 45-55 years) (Flurkey et al., 2007). Analysis of body weight (Fig. S2A) and the weight of different skeletal muscles [tibialis anterior, gastrocnemius, quadriceps, extensor digitorum longus (EDL) and soleus muscles; Fig. S2B-F] showed a significant body and muscle weight decrease in the chronic MCM/FLExD-mature and -aged animals compared with age-matched *ACTA1-MCM/+* controls. Histological analysis of DUX4 immunostaining revealed the presence of scattered DUX4-positive fibers in 1-month-old muscle and a gradual qualitative increase in the number of DUX4-positive nuclei in chronic MCM;FLExD-young, -mature and -aged muscles (Fig. 3A-P). Although quantitative analysis of DUX4 protein expression by immunostaining was hindered by frequent high background staining in the myofibers and additional unspecific staining in interstitial cells, potentially immune cells, in MCM;FLExD-mature and -aged animals, overall, we observed that the number of recombined DUX4-expressing myonuclei increased over time, consistent with recent data that endogenous DUX4 expression is not immediately cytotoxic in FSHD myogenic cells (Chau et al., 2021). Similarly, *DUX4-fl* levels increased significantly as the *ACTA1-MCM;FLExD/+* mice aged. Young, mature and aged mice displayed significantly higher *DUX4-fl* expression compared with age-matched *ACTA1-MCM/+* controls (Fig. 3Q); however, it should be noted that *DUX4-fl* levels were highly variable in chronic MCM;FLExD-mature and -aged muscles, which needs to be considered in downstream analyses (Fig. 3). Overall, *DUX4-fl* levels progressively increased with age in the bi-transgenic *ACTA1-MCM;FLExD/+* mice, indicating that chronic expression of *DUX4* leads to a cumulative increase in DUX4-positive myonuclei and *DUX4* mRNA levels, suggesting that many aged DUX4-positive myofibers were accumulating, rather than dying (Fig. 3).

**Fig. 4.**
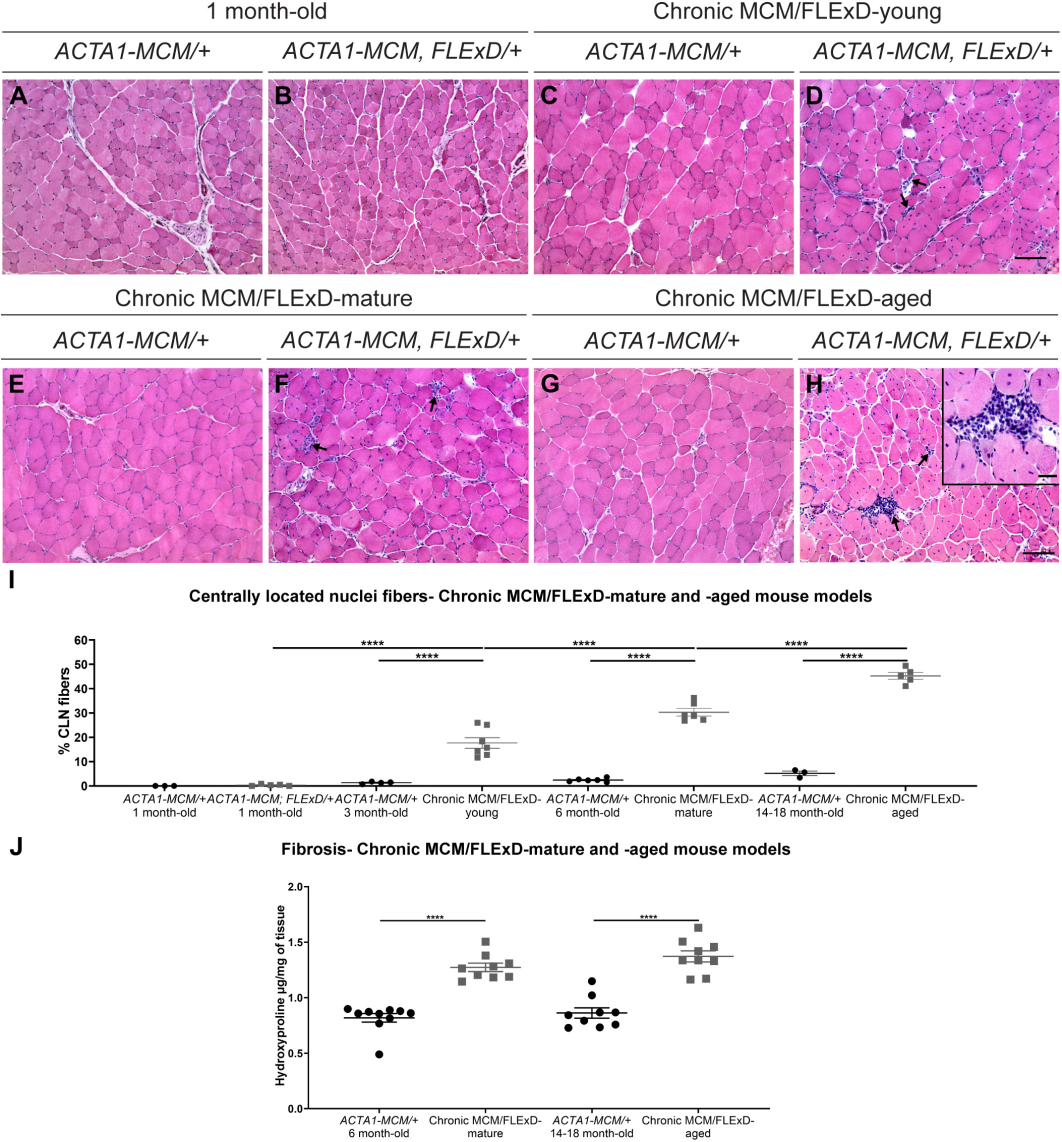
**Histological analysis of the chronic DUX4 mouse models reveals FSHD-like histopathological changes in skeletal muscle.** (A-H) Hematoxylin and Eosin staining of gastrocnemius muscles from 1-month-old MCM/FLExD mice (A,B), chronic MCM/FLExD-young mice (C,D), chronic MCM/FLExD-mature mice (E,F) and chronic MCM/FLExD-aged mice (G,H), compared with age-matched control *ACTA1-MCM/+* mice. Arrows show immune cell infiltration marked by interstitial aggregation of mononuclear cells, which is often observed with myofiber necrosis. High-magnification image inset in H scale bar: 10 µm. Representative images are shown for each experimental group (*n*=3 animals). Scale bars: 100 µm. (I) Quantification of centrally localized nuclei-positive myofibers in gastrocnemius muscles isolated from 1-month-old, chronic MCM/FLExD-young, chronic MCM/FLExD-mature and chronic MCM/FLExD-aged mice compared with age-matched control *ACTA1-MCM/+* mice. Statistical analysis was performed with two-tailed one-way ANOVA (uncorrected Fisher's LSD) with *n*=3 animals for *ACTA1-MCM/+* 1-month-old, *n*=5 animals for *ACTA1-MCM;FLExD/+* 1-month-old, *n*=4 animals for *ACTA1-MCM/+* 3-month-old, *n*=7 animals for chronic MCM/FLExD-young, *n*=6 animals for *ACTA1-MCM/+* 6-month-old, *n*=6 animals for chronic MCM/FLExD-mature, *n*=3 animals for *ACTA1-MCM/+* 14- to 18-month-old and *n*=6 animals for chronic MCM/FLExD-aged experimental groups. (J) Fibrosis was analyzed by hydroxyproline quantification in gastrocnemius muscles isolated from 1-month-old, chronic MCM/FLExD-young, chronic MCM/FLExD-mature and chronic MCM/FLExD-aged mice compared with age-matched control *ACTA1-MCM/+* mice. Statistical analysis was performed with two-tailed one-way ANOVA (uncorrected Fisher's LSD) with *n*=10 animals for *ACTA1-MCM/+* 6-month-old, *n*=9 animals for chronic MCM/FLExD-mature, *n*=9 animals for *ACTA1-MCM/+* 14- to 18-month-old and *n*=9 animals for chronic MCM/FLExD-aged experimental groups. Data are presented as mean±s.e.m.; ****P<0.0001.

**Fig. 5.**
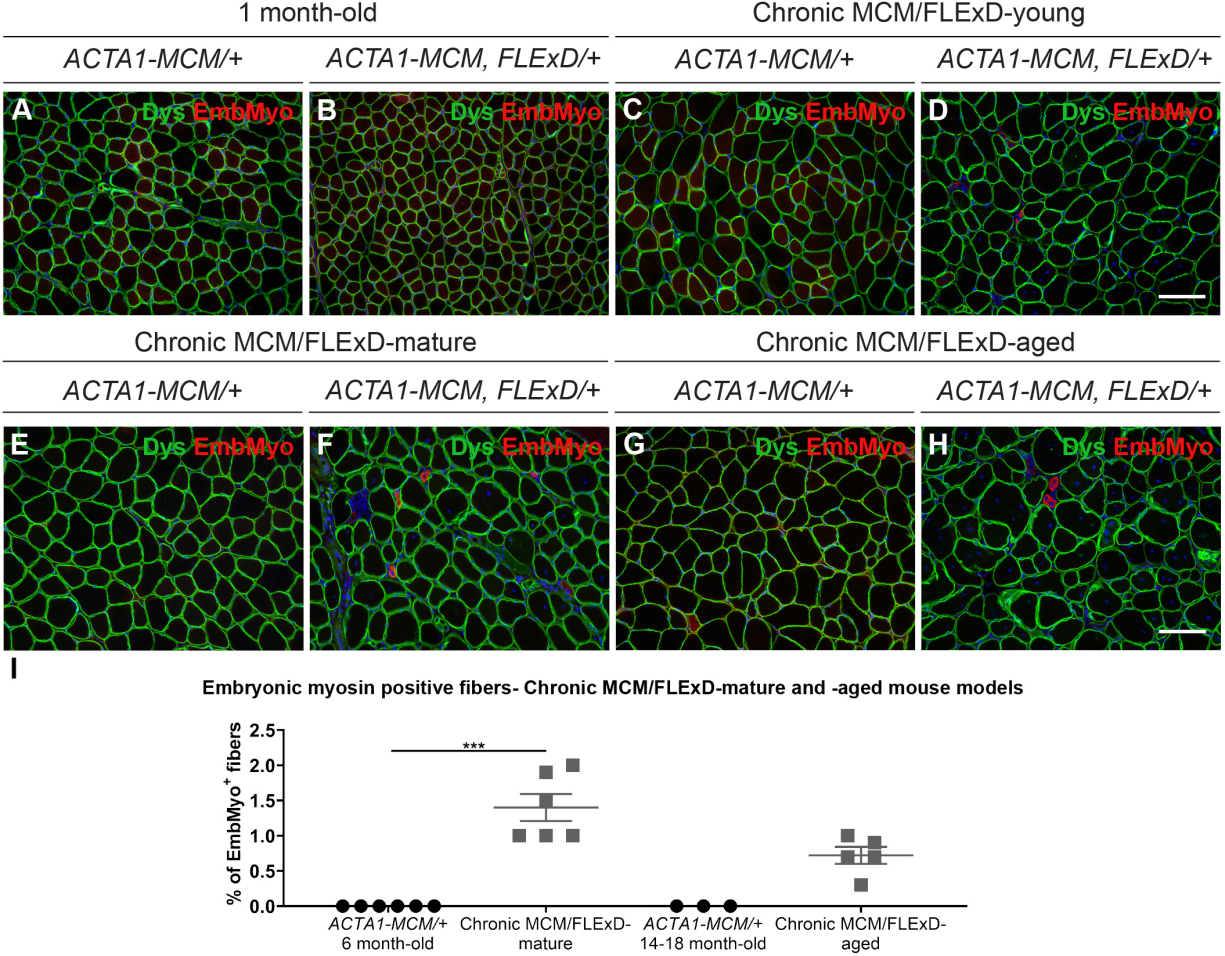
**Histological analysis of muscle regeneration in the chronic DUX4 mouse models.** (A-H) Immunofluorescence staining for dystrophin (green), embryonic myosin (red) and DAPI (blue) in gastrocnemius muscles from 1-month-old *MCM/FLExD* mice (A,B), chronic MCM/FLExD-young mice (C,D), chronic MCM/FLExD-mature mice (E,F) and chronic MCM/FLExD-aged mice (G,H), compared with age-matched control *ACTA1-MCM/+* mice. Representative images are shown for each experimental group (*n*=3 animals). Scale bars: 100 µm. (I) Quantification of embryonic myosin-positive myofibers in gastrocnemius muscles isolated from chronic MCM/FLExD-mature mice and chronic MCM/FLExD-aged mice compared with age-matched control *ACTA1-MCM/+* mice. Statistical analysis was performed with two-tailed Kruskal–Wallis test (no Dunn's test) with *n*=6 animals for *ACTA1-MCM/+* 6-month-old, *n*=6 animals for chronic MCM/FLExD-mature, *n*=3 animals for *ACTA1-MCM/+* 14- to 18-month-old and *n*=5 animals for chronic MCM/FLExD-aged experimental groups. Data are presented as mean±s.e.m.; ***P<0.001.

**Fig. 6.**
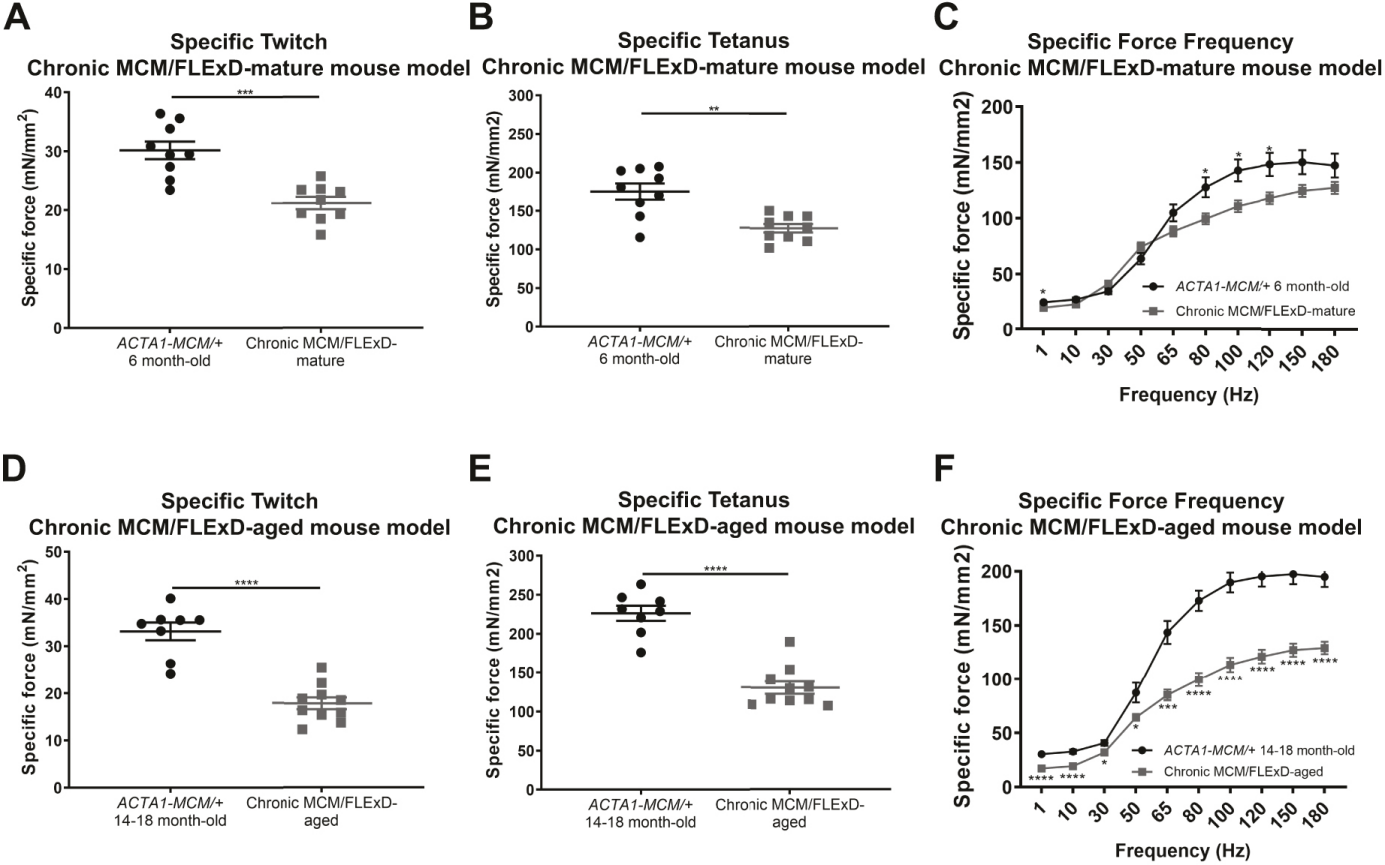
***Ex vivo* physiology analysis of extensor digitorum longus in chronic DUX4-expressing FSHD-like mouse models.** (A-F) Physiology measurements for specific twitch (A,D), specific tetanus (B,E) and force frequency (C,F) in the chronic MCM/FLExD-mature and -aged mouse model. Statistical analysis was performed with two-tailed Welch's *t*-test and two-way ANOVA with *n*=9 animals for *ACTA1-MCM/+* 6-month-old, *n*=9 animals for chronic MCM/FLExD-mature, *n*=8 animals for *ACTA1-MCM/+* 14- to 18-month-old and *n*=10 animals for chronic MCM/FLExD-aged experimental groups. Data are presented as mean±s.e.m.; *P<0.05, **P<0.01, ***P<0.001, ****P<0.0001.

To assess the effects of chronic, low, mosaic DUX4 expression on skeletal muscle pathology, we analyzed muscles by immunofluorescence and Hematoxylin and Eosin (H&E) staining (Figs 4 and 5). Histological analysis of the gastrocnemius muscle revealed that chronic exposure to *DUX4* leads to a steady increase in the percentage of damaged/regenerated fibers marked by centrally localized nuclei from 0% to 45%, and also an increase in immune cell infiltration over a period of 1.5 years. Control *ACTA1-MCM/+* muscles did not show any significant increase in regenerated myofibers or immune cell infiltration over the same period (Fig. 4A-H,I). An equivalent significant increase in centrally localized nuclei was detected in the tibialis anterior muscle (Fig. S2G-K). Furthermore, fibrosis levels, measured by the muscle hydroxyproline content, were significantly increased in MCM;FLExD-mature and -aged muscles compared with age-matched *ACTA1-MCM/+* controls (Fig. 4J). This significant increase in the percentage of fibers with centralized nuclei and fibrosis in chronic MCM;FLExD-aged muscles compared with chronic MCM;FLExD-mature muscles correlates with an increase in DUX4 expression (Fig. 3 and Fig. 4I,J). However, analysis of embryonic myosin, a marker for muscle regeneration, found that the percentage of embryonic myosin-positive fibers in both older populations of *ACTA1-MCM;FLExD/+* DUX4-expressing muscles corresponded, on average, to <2% of the total number of fibers (Fig. 5A-H,I). This suggests that chronically increased levels of DUX4 expression lead to sporadic muscle regeneration events, as opposed to massive muscle regeneration, and, ultimately, to cumulative muscle pathology. We then analyzed muscle physiology of the EDL muscle from the chronic MCM/FLExD-mature and -aged mice (Fig. 6). As expected, chronic MCM;FLExD-mature and -aged muscles displayed significantly lower strength in specific twitch (Fig. 6A,D), tetanus (Fig. 6B,E), and force-frequency (Fig. 6C,F) measurements compared with age-matched *ACTA1-MCM/+* controls. Taken together, these results indicate that aged *ACTA1-MCM;FLExD/+* muscles exposed to chronic, low levels of DUX4 develop a complex pathology that more accurately recapitulates FSHD than previous models.

### Immune/regeneration-related muscle miRNA profile in chronic DUX4-expressing FSHD-like mouse models

**Fig. 7.**
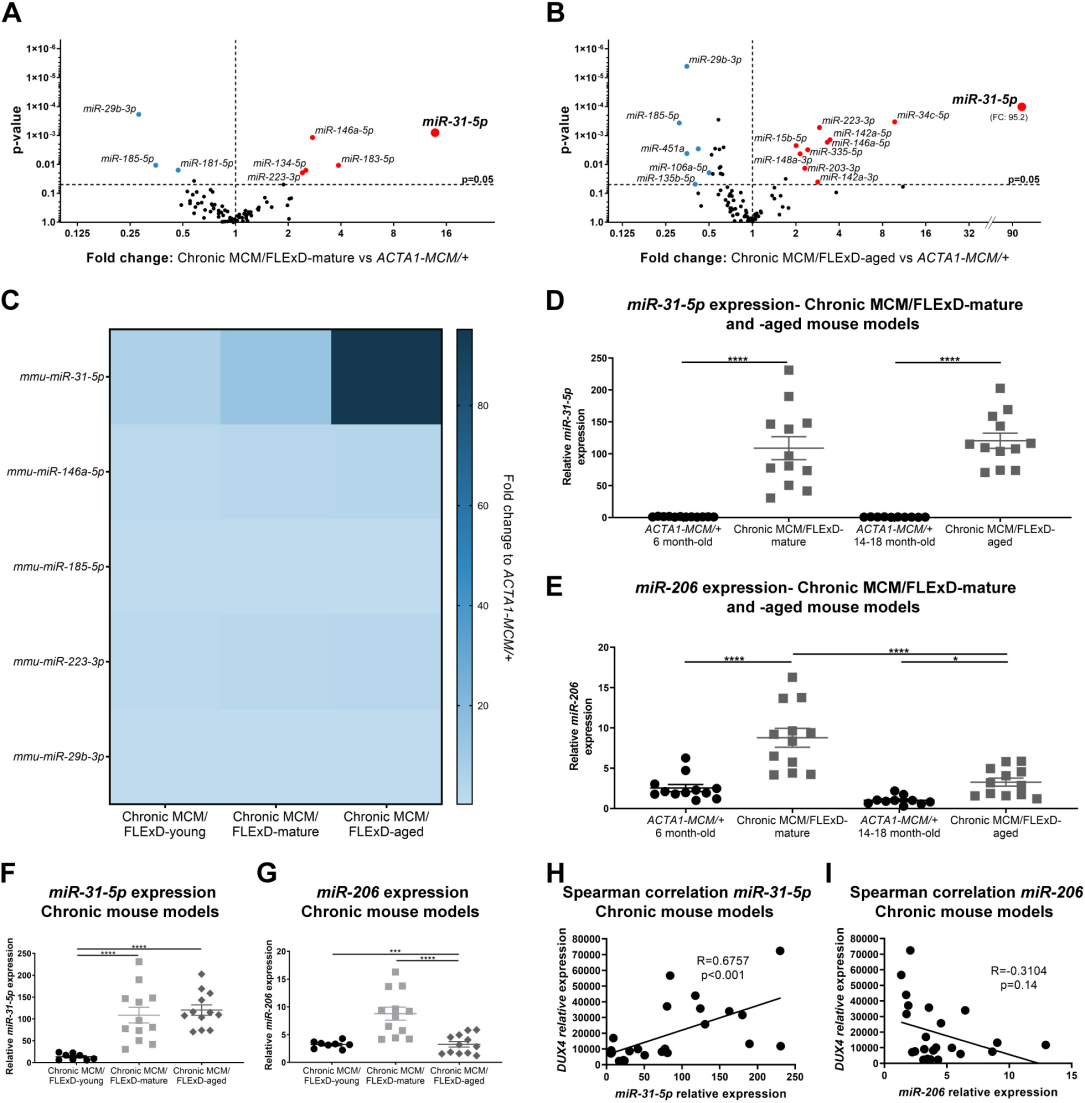
**Targeted miRNA profile in the chronic DUX4 FSHD-like mouse models shows dysregulation of miRNAs in skeletal muscle.** (A,B) Volcano plots depict the miRNA profile in gastrocnemius muscle from the chronic MCM/FLExD-mature model (A) and the chronic MCM/FLExD-aged model (B) compared with age-matched control *ACTA1-MCM/+* mice (*n*=6-9 animals for each experimental group). Red and blue circles depict miRNAs for which levels are significantly upregulated and downregulated by ≥2-fold, respectively. Volcano plots represent two-tailed unpaired Student's *t*-test analysis with *n*=7 animals for *ACTA1-MCM/+* 6-month-old, *n*=9 animals for chronic MCM/FLExD-mature, *n*=6 animals for *ACTA1-MCM/+* 14- to 18-month-old and *n*=8 animals for chronic MCM/FLExD-aged experimental groups. (C) Heat map for significantly dysregulated miRNAs in the gastrocnemius muscle isolated from the chronic MCM/FLExD-young, -mature and -aged mouse models (*n*=6-9 for each group). (D,E) qRT-PCR quantification of *miR-31-5p* (D) and *miR-206* (E) in gastrocnemius muscle isolated from chronic MCM/FLExD-mature and chronic MCM/FLExD-aged mouse models. (F,G) Combined expression profiles for *miR-31-5p* (F) and *miR-206* (G) in the chronic MCM/FLExD-young, -mature and -aged mouse models. Statistical analysis was performed with two-tailed one-way ANOVA test (uncorrected Fisher's LSD) with *n*=8 animals for chronic MCM/FLExD-young, *n*=12 animals for *ACTA1-MCM/+* 6-month-old, *n*=12 animals for chronic MCM/FLExD-mature, *n*=10 animals for *ACTA1-MCM/+* 14- to 18-month-old and *n*=12 animals for chronic MCM/FLExD-aged experimental groups. Data are presented as mean±s.e.m.; *P<0.05, ***P<0.001, ****P<0.0001. (H,I) Spearman correlation for *miR-31-5p* (H) or *miR-206* (I) and *DUX4* expression in gastrocnemius muscle from the chronic MCM/FLExD-mature and -aged mouse models.

Late-onset and progressive muscle pathology in FSHD patients is thought to be initiated by chronic, low, sporadic expression of *DUX4* throughout one's lifetime (Haynes et al., 2018; Jones et al., 2012; Kowaljow et al., 2007; Rickard et al., 2015; Snider et al., 2010; Tassin et al., 2013). Because we found that our chronic DUX4-expressing mouse model also develops late-onset, accumulative FSHD-like pathology including immune infiltration, we further investigated the immune/regeneration-related miRNA profile in the later phases of pathogenesis after 1.5 years of exposure to chronic DUX4 expression in skeletal muscle. We analyzed gastrocnemius muscle from the chronic MCM;FLExD-mature and -aged mouse models (Fig. 7; Fig. S1B, Table S1) and found six notable categories of dysregulated miRNAs: category 1, miRNAs for which upregulation correlates with pathogenesis progression (*miR-31-5p* and *miR-146a-5p*); category 2: miRNAs for which upregulation throughout pathogenesis does not correlate with pathology severity (*miR-206*); category 3, miRNAs upregulated during the early to mid phase of pathogenesis (*miR-183-5p*); category 4, miRNAs upregulated during the mid to late phase of pathogenesis (*miR-223-3p*); category 5, miRNAs upregulated in the late phase of pathogenesis (*miR-15b-5p*, *miR-148a-3p*, *miR-335-5p*, *miR-203-3p*); and category 6, miRNAs downregulated throughout pathogenesis (*miR-29b-3p* and *miR-185-5p*). Overall, the levels of immune-related miRNAs were consistently increased in chronic MCM;FLExD muscles compared with age-matched control, whereas the levels of *miR-29b-3p*, a hallmark of atrophic muscles (Hu et al., 2014), were consistently downregulated (Fig. 7A,B).

Perhaps the most interesting finding in this miRNA analysis of chronic DUX4-expressing FSHD mouse models was that *miR-31-5p*, the levels of which were altered in the short-term DUX4-induced moderate model, was in category 1. Strikingly, *miR-31-5p* expression levels were steeply upregulated by 6-fold, 14-fold and 95-fold in chronic MCM;FLExD-young, -mature and -aged muscles, respectively (Fig. 1A and Fig. 7A-C). TaqMan probe quantification of *miR-31-5p* confirmed that its levels were significantly increased by 97-fold, on average, in chronic MCM;FLExD-mature muscles and by 139-fold in chronic MCM;FLExD-aged muscles compared with *ACTA1-MCM/+* control muscles (Fig. 7D,F), and significantly higher than in muscles from the chronic MCM;FLExD-young mice (Fig. 2B and Fig. 7F). Interestingly, *miR-31-5p* levels were significantly different in females and males in the chronic MCM/FLExD-mature animals, but this difference was not detected in the older chronic MCM/FLExD-aged animals (Fig. S3A). To determine whether *miR-31-5p* levels correlate with *DUX4-fl* levels in chronic DUX4-expressing muscles, we tested for potential associations between *DUX4-fl* and *miR-31-5p* expression using Spearman's correlation. We found a significant correlation between *miR-31-5p* and *DUX4-fl* expression, suggesting that dysregulation of *miR-31-5p* is DUX4 dependent and may be DUX4 responsive (Fig. 7H). Regardless, the strongly increased upregulation of *miR-31-5p* over the progressive FSHD-like pathogenesis in this chronic DUX4 model suggests that *miR-31-5p* is a potential FSHD disease biomarker for muscle biopsy. We also assessed *miR-206* in the chronic DUX4-expressing FSHD-like mouse models, as this was a promising candidate biomarker from our analysis using the DUX4-induced *ACTA1-MCM;FLExD/+* models. We found that the levels of *miR-206*, as determined by TaqMan qRT-PCR, were also significantly increased in gastrocnemius muscle from chronic MCM/FLExD-mature and -aged muscles by 7- and 3-fold, respectively, compared with chronic MCM/FLExD-young muscles (Fig. 7E,G). *miR-206* expression was similar in females and males from chronic MCM/FLExD-mature and -aged models (Fig. S3B). We then tested for potential associations between *miR-206* and *DUX4-fl* expression levels using Spearman's correlation, but did not find a significant correlation (Fig. 7I). Nevertheless, because *miR-31-5p* and *miR-206* levels are increased in skeletal muscle throughout the course of DUX4-induced pathogenesis, they are very attractive potential FSHD biomarkers for further evaluation.

### *miR-206* is a candidate FSHD serum biomarker

**Fig. 8.**
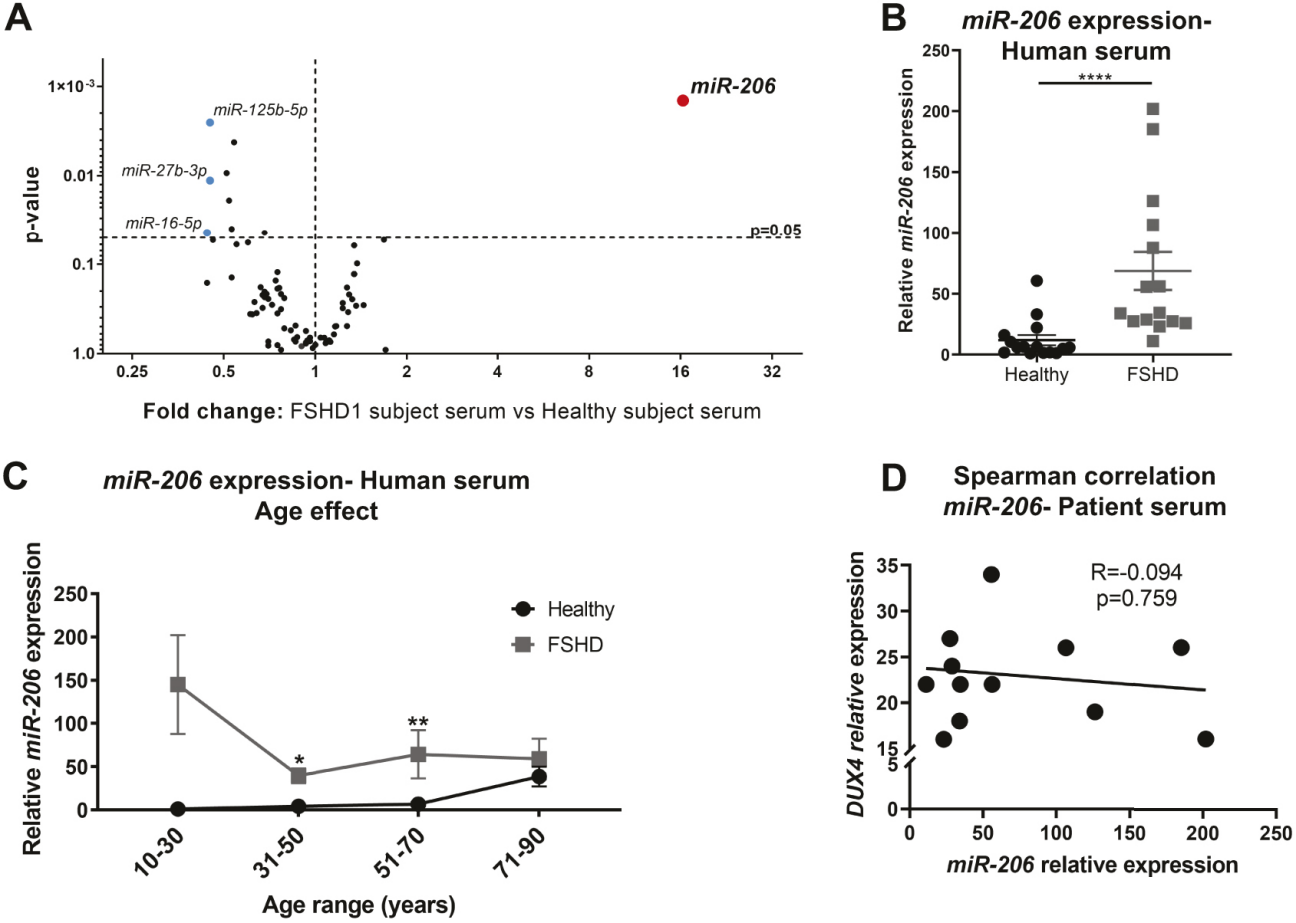
**Expression of *miRNA-206* is significantly upregulated in FSHD patient serum.** (A) Volcano plot for the miRNA profile in FSHD patient serum (*n*=7 FSHD patients and *n*=10 healthy subjects). Red and blue circles depict miRNAs for which levels are significantly upregulated and downregulated by ≥2-fold, respectively. Volcano plots depict two-tailed unpaired Student's *t*-test analysis. (B) *miR-206* expression in human serum from all patients in the study (*n*=14 FSHD patients and *n*=15 healthy subjects). Statistical analysis was performed using the two-tailed Mann–Whitney *U*-test with *n*=15 FSHD patients and *n*=15 healthy subjects. (C) *miR-206* expression in human serum depicted according to patient age. Statistical analysis was performed using the two-tailed Mann–Whitney *U*-test with *n*=15 FSHD patients and *n*=15 healthy subjects. Data are presented as mean±s.e.m.; *P<0.05, **P<0.01, ****P<0.0001. (D) Spearman correlation of *miR-206* expression and *DUX4* D4Z4 reduced allele size (*Eco*RI/*Bln*l-kb) with *n*=14 FSHD1 patients.

Discovery work using the above mouse models identified a number of DUX4-dependent miRNAs that are attractive candidates for FSHD biomarkers. However, although these were identified from muscle tissue, the goal of this study was to identify circulating biomarkers for FSHD for non-invasive assessment in clinical trials. Therefore, the candidate miRNA profiles were validated using serum samples isolated from FSHD patients and healthy controls. We collected serum samples from 15 healthy subjects and 15 clinically affected and genetically confirmed FSHD patients and processed these samples for the equivalent human immunopathology miRNA profile used for the murine muscle samples (Fig. 8; Table S2). Surprisingly, we found that *miR-206* was the only miRNA significantly upregulated in FSHD patient serum compared with serum from healthy subjects (Fig. 8A). Further analysis by TaqMan probe assay confirmed that although *miR-206* levels were highly variable in the FSHD study population, they were nonetheless significantly elevated in FSHD patient serum (Fig. 8B). Because *miR-206* expression is known to increase with age in healthy mice (Hamrick et al., 2010), and inclusion of older subjects could therefore bias the analysis of biomarkers, we stratified the study population by age and verified that the significant increase in *miR-206* levels in serum is only significant in the 31-50 and 51-70 age-range groups (Fig. 8C). Furthermore, *miR-206* expression did not correlate with age or D4Z4 deletion size (Fig. 8D), just FSHD clinical status. An equivalent analysis of mouse serum revealed that the miRNA levels in serum are too low for a reliable quantification (>30 Cq considered unreliable for analysis of pre-amplified products by the manufacturer) (Fig. S4). Overall, these data support that *miR-206* is a strong candidate DUX4-dependent circulating biomarker for the presence of muscle damage such at that found in clinical FSHD.

## DISCUSSION

Identifying reliable and independently validated circulating DUX4-dependent biomarkers is one of the great unmet needs in the FSHD field, as none have been described to date. The discovery of circulating serum biomarkers could greatly impact the prediction of disease progression and the determination of therapeutic efficacy in clinical trials, and thus is of utmost need. With the recent development of FSHD-like mouse models (Bosnakovski et al., 2017; Giesige et al., 2018; Jones and Jones, 2018; Jones et al., 2018 preprint), research on FSHD disease biomarkers has become technically more feasible. Here, we describe new aged FSHD-like mouse models of chronic DUX4 expression that recapitulate many important aspects of FSHD pathology and characterize the muscle miRNA signature in these models. We demonstrate that *miR-31-5p* and *miR-206* levels are consistently increased in the muscle of FSHD-like mouse models and that *miR-206* expression is significantly increased in serum samples from FSHD patients. We propose *miR-206* as a new candidate circulating disease biomarker for FSHD.

### Chronic DUX4-expressing FSHD-like mouse model

Although multiple strains of inducible FSHD-like mouse models have now been generated (Bosnakovski et al., 2017, 2020; Giesige et al., 2018; Jones and Jones, 2018; Jones et al., 2020), these either rely on acute induction of DUX4 to produce massive pathology or on long-term small-molecule treatments resulting in chronic expression of DUX4 in both muscle and non-muscle tissues. None of these models mimics the typical situation in FSHD in which pathology is caused by sporadic, but chronic, expression of DUX4 in skeletal muscles throughout one's lifetime (Haynes et al., 2018; Jones et al., 2012; Rickard et al., 2015; Snider et al., 2010; Tassin et al., 2013). Here, we characterized two aged models recapitulating chronic skeletal muscle-specific DUX4 expression: one at 6 months old (chronic MCM;FLExD-mature model) and one at 14-18 months old (chronic MCM;FLExD-aged model) using the *ACTA1-MCM;FLExD/+* mice. Importantly, generation of these chronic FSHD-like models does not require any treatments to activate the chronic expression of DUX4 in either male or female animals and thus provides an important advantage for consistency and reproducibility in long-term studies. We demonstrated that DUX4 expression increases progressively in chronic MCM;FLExD-young, -mature and -aged muscles compared with 1-month-old asymptomatic muscles and age-matched control *ACTA1-MCM/+* muscles. The data also revealed that chronic DUX4 expression induced hallmarks of FSHD pathology such as increased centrally nucleated myofibers (damaged, regenerated myofibers), immune infiltration and reduced muscle regeneration (Arahata et al., 1995; Banerji et al., 2020; Frisullo et al., 2011; Honda et al., 1987; Padberg, 1982; Statland et al., 2015b). Accordingly, immunostaining for DUX4 protein in the muscles of chronic FSHD-like mice revealed the presence of unspecific staining in interstitial cells, possibly immune cells, and prevented an accurate quantification. In addition, this suggests that the muscle from these mice is more immunoreactive than that from the age-matched controls.

In contrast to most other muscular dystrophies such as DMD, in which the underlying primary defects cause structural myofiber disruption, leading to high levels of degeneration and regeneration, FSHD is a slowly progressive disease caused by expression of a toxic protein, DUX4, exclusively in a very few myonuclei. The ultimate result is the development of a myopathy with many general hallmarks of muscle degeneration that coincide with the other muscular dystrophies, but also with several features that are distinct to FSHD as well. That is evidenced by differences in muscle regeneration levels as assessed by embryonic myosin staining. Whereas DMD muscle biopsies display 24-33% regenerating fibers (Janghra et al., 2016; Schiaffino et al., 1986), FSHD muscle biopsies exhibit only 0.48% and 1.72% regenerating fibers in the quadriceps and tibialis anterior, respectively (Banerji et al., 2020). This strikingly low level of regeneration is also common to myotonic muscular dystrophy type 2, another slowly progressing muscle disease (Banerji et al., 2020). Thus, our chronic FSHD-like mouse model better recapitulates both the skeletal muscle-specific mosaic expression of DUX4 and the hallmark pathophysiology of FSHD. It is important when using mouse models for biomarker discovery and investigations into disease mechanisms to minimize the interference of tissues not affected in the human condition as well as to avoid degeneration/regeneration-related artifacts. For comparison, an alternative transgenic mouse model for chronic FSHD has recently been proposed; however, unlike in the human disease situation, the model uses constitutive tetracycline treatment to induce DUX4 at low levels in both muscle and non-muscle tissues of female mice (Bosnakovski et al., 2020). By contrast, our aged chronic FSHD-like mouse models based on the *ACTA1-MCM;FLExD/+* bi-transgenic animals more accurately recapitulate FSHD pathogenic mechanisms and pathophysiology, and are thus ideally suited to identify disease biomarkers, study potential disease mechanisms and determine the effects of potential therapeutics.

### DUX4-induced miRNA dysregulation in FSHD-like mouse models

miRNA profiles can be informative in understanding both healthy and pathogenic mechanisms as well as revealing biomarkers for disease. For example, immune-related miRNAs such as *miR-146b*, *miR-155*, *miR-214*, *miR-221* and *miR-222* are consistently found to be dysregulated in the muscular dystrophies, including FSHD (Eisenberg et al., 2007). Portilho et al. (2015) identified *miR-330*, *miR-331-5p*, *miR-34a*, *miR-380-3p*, *miR-516b*, *miR-582-5p*, *miR-517* and *miR-625* as being exclusively expressed in human FSHD1 quadriceps biopsies obtained from fetuses compared with healthy muscles. However, Heier et al. (2020) recently identified a very different set of eight miRNAs in FSHD patient serum, with only *miR-34a* and *miR-146b* overlapping with either the Portilho et al. (2015) or Eisenberg et al. (2007) study, respectively. Thus, there has been great variability between studies on miRNAs using different types of FSHD clinical samples, and no studies have shown any miRNA as being DUX4 dependent.

Here, we used DUX4-expressing, FSHD-like model mice to identify DUX4-dependent dysregulated miRNAs in skeletal muscle. The data revealed that multiple immune-related miRNAs were dysregulated, supporting a role for the immune response in contributing to DUX4-dependent FSHD muscle pathology and degeneration. Interestingly, none of these miRNAs were significantly dysregulated in the serum from FSHD patients, which suggests that these immune-related miRNAs are part of a local response to muscle damage. This is consistent with a recent study that reported dysregulation of *miR-142-3p* and *miR-146b* levels in mildly affected FSHD patients, but failed to show significance upon larger validation (Heier et al., 2020).

The most strikingly dysregulated miRNA in both of our models is *miR-31-5p*, which is expressed in immune cells as well as in regenerating skeletal muscle (Cacchiarelli et al., 2011a; Greco et al., 2009; Alrafas et al., 2020; Xue et al., 2013; Zhu et al., 2019). Whether the DUX4-dependent increase in *miR-31-5p* levels takes place in DUX4-expressing myofibers or in infiltrating non-muscle cells has yet to be determined. If the dysregulation takes place in myofibers, *miR-31-5p* may serve as a useful interstitial muscle fluid biomarker of disease. Considering that muscle microdialysis was already successfully implemented in the study of muscle fluid from FSHD patients (Tasca et al., 2017), this alternative methodology holds a promising new avenue for FSHD human muscle analysis. Although *miR-31-5p* levels are sensitive to DUX4 expression in muscle, this miRNA may not be a direct target of DUX4. The potential involvement of *miR-31-5p* in DUX4 downstream cascades is supported by the dysregulation of *miR-31-5p* in several inflammatory-related disorders such as cancer (Cho et al., 2015; Necela et al., 2011; Yi et al., 2019), psoriasis (Abdallah and Pichon, 2019; Xu et al., 2013), experimental autoimmune encephalomyelitis (Zhang et al., 2015), inflammatory bowel disease (Olaru et al., 2011), lupus (Dai et al., 2010) and asthma (Shi et al., 2019). In particular, *miR-31-5p* is an important regulator of cytokine/chemokine production (Dai et al., 2019; Xu et al., 2013) and neutrophil function and T-cell activation during inflammation (Alrafas et al., 2020; Rovira-Llopis et al., 2018; Suárez et al., 2010; Xue et al., 2013), and is dysregulated in the *mdx* mouse model and muscle biopsies from DMD patients (Cacchiarelli et al., 2011a; Fiorillo et al., 2015), likely as a consequence of its high expression in regenerating fibers (Cacchiarelli et al., 2011a; Greco et al., 2009). However, the low levels of muscle regeneration in our FSHD-like mouse models suggest that the role of *miR-31-5p* in response to DUX4 expression may be different from that in *mdx*/DMD. As with the other immune-related miRNAs, *miR-31-5p* levels were not dysregulated in FSHD patient serum, similar to previous findings (Heier et al., 2020), again suggesting that this dysregulation may be a local response. Further studies will be important to clarify whether *miR-31-5p* levels are elevated in FSHD patient biopsies and in which cells *miR-31-5p* levels are upregulated.

### *miR-206* is a potential new disease biomarker for FSHD

Several miRNAs have been described as circulating biomarkers for neuromuscular diseases such as amyotrophic lateral sclerosis (Si et al., 2018; Toivonen et al., 2014) and DMD (Cacchiarelli et al., 2011b; Mizuno et al., 2011; Roberts et al., 2012, 2013), and unrelated diseases such as cancer (Wang et al., 2014). Yet, although miRNA profiling in the current study revealed the dysregulation of multiple immune-related miRNAs in DUX4-expressing muscle, none of these were validated as circulating FSHD biomarkers. Interestingly, the levels of *miR-206*, one of the original myomiRs and a major regulator of muscle myogenesis and regeneration (Chen et al., 2010; Greco et al., 2009; Horak et al., 2016; Kim et al., 2006; Ma et al., 2015; Yuasa et al., 2008), were consistently increased in the muscle from all FSHD-like mouse models and in serum from FSHD patients. Although *miR-206* levels were found to be similar in muscle biopsies from healthy and FSHD fetuses (Portilho et al., 2015), this is not necessarily at odds with our findings in adult FSHD-like mice. Because *miR-206* is a major regulator of myogenesis, the mechanisms controlling its expression as well as its DUX4 dependence are likely to be very different in developing versus adult muscle. In fact, *miR-206* levels progressively increase in later stages of human fetal skeletal muscle development, suggesting that *miR-206* has a different impact during different phases of skeletal muscle development *in utero*, and its dynamic profile has to be taken into consideration when comparing developing and adult muscle (Koutsoulidou et al., 2011). Additionally, although a previous study reported that *miR-206* levels were unchanged in FSHD muscle biopsies (Eisenberg et al., 2007), incongruities regarding miRNA profiles have been reported previously in DMD and, as discussed previously, technical differences might account for differences in the reported miRNA signatures (Roberts et al., 2012). Our current study relies on a miRNA array profile further validated by TaqMan qRT-PCR quantification and thus provides consistent support for *miR-206* dysregulation in mouse muscle biopsies and human serum samples. Unfortunately, the most recent report on FSHD circulating biomarkers did not include *miR-206* in the analysis (Heier et al., 2020). Thus, further independent studies still need to be performed and will be important to validate *miR-206* as a circulating FSHD biomarker. Nonetheless, *miR-206* has been proposed as a serum biomarker for DMD and DM1 (Myotonic dystrophy type 1) (Cacchiarelli et al., 2011b; Koutsoulidou et al., 2015, 2017; Mizuno et al., 2011; Roberts et al., 2012, 2013), and we propose it as a strong candidate for FSHD as well.

Taken together, we describe two new FSHD-like mouse models of chronic DUX4 expression that recapitulate important hallmarks of FSHD gene expression and cumulative pathology. We provide a thorough characterization of the miRNA profile in muscles of these models, as well as in serum samples from FSHD patients. We identified *miR-31-5p* and *miR-206* as prospective regulators of muscle disease in response to DUX4 expression and validated *miR-206* as a candidate DUX4-dependent serum biomarker for FSHD. Future studies on the mechanisms underlying the involvement of *miR-206* in FSHD pathology, as well as independent analyses of larger cohorts of FSHD patients and healthy controls, will be important for further validation of this miRNA as a useful clinical biomarker for FSHD.

## MATERIALS AND METHODS

### Human patients

This project was approved by the University of Nevada, Reno Institutional Review Board (#1316095). Demographic and clinical information are provided in Table S2. This clinical investigation was conducted according to the principles expressed in the Declaration of Helsinki. All patient sera were de-identified and processed in a randomized and blinded manner.

### Animals

All procedures involving animals were approved by University of Nevada, Reno Institutional Animal Care and Use Committee (protocol #20-09-1078). The care and use of experimental animals complied with relevant institutional and national animal welfare laws, guidelines and policies. The *ACTA1-MCM* line (McCarthy et al., 2012) was purchased from The Jackson Laboratory (Bar Harbor, ME, USA). *ACTA1-MCM;FLExD* mice were generated and genotyped as previously described (Jones et al., 2020). *ACTA1-MCM;FLExD* mice were injected with one 5 mg/kg dose of TMX, as described (Jones et al., 2020), to create the moderate FSHD-like model, and were analyzed at day 3, 9 and 28 after TMX administration. Mice designated as 1 and 3 months old correspond to 4- and 13- to 14-week-old mice, respectively. Mice were sacrificed under deep anesthesia (3% isoflurane). Analysis of mouse tissue was performed in a blinded manner.

### Total RNA extraction

Gastrocnemius muscles were freshly dissected and immediately snap-frozen in liquid nitrogen. A portion of gastrocnemius mid belly was collected for total RNA (including small RNAs) extraction with an RNeasy Mini Kit as indicated by the manufacturer (Qiagen, 74104). Briefly, muscle was homogenized in ten times the volume of the sample using Trizol reagent in a tissue lyser for 10 min at 50 Hz. The lysates were then incubated at room temperature for 7 min and RNA was separated with 0.2× total volume of chloroform (room temperature incubation for 3 min). After phase separation (12,000 ***g*** for 15 min at 4°C), the aqueous phase was mixed with 1.5× total volume of 100% ethanol. The sample was then transferred to an RNeasy MinElute column and centrifuged for 30 s at 10,000 ***g***. The column was then washed with Buffer RWT and treated with DNase. After a second Buffer RWT wash, the column was washed twice with Buffer RPE and twice with 80% ethanol. The RNeasy MinElute column was dried for 5 min, and RNA was eluted with RNase-free water. Mouse and human serum samples were collected after blood centrifugation at 1000 ***g*** for 10 min and 15 min, respectively, at 15°C. Total RNA (including small RNAs) was extracted from 500 µl human serum and 200 µl mouse serum with the miRNeasy Serum/Plasma Advanced Kit, as indicated by the manufacturer (Qiagen, 217204).

### Reverse transcription and quantitative PCR (qPCR) reactions for miScript profile

Reverse transcription reaction was performed according to the manufacturer's instructions with 250 ng RNA (Qiagen, 218161). The reverse transcription reaction mixture was then diluted in 200 µl RNase-free water and 100 µl of the resulting solution was subsequently used for the qPCR reaction. miScript miRNA profiles for the immunopathology panel (MIMM-104ZD for mouse, and MIHS-104ZD for human) were prepared with SYBR Green (Qiagen, 218076) according to the manufacturer's instructions in a 96-well plate (Qiagen, 218076). The cycling conditions were set up as indicated by the manufacturer. All samples were compared after baseline cycle and threshold value standardization. RNA levels were normalized to Snord68/SNORD68 (Small Nucleolar RNA, C/D Box 68) (Zanotti et al., 2018) and global Ct mean for mouse gastrocnemius muscle and human serum, respectively.

### TaqMan qRT-PCR analysis

The reverse transcription reaction was performed with 10 ng total RNA from gastrocnemius muscle and 3 µl serum according to the manufacturer's instructions (Thermo Fisher Scientific, 4366596). Pre-amplification was performed for serum-extracted RNA (Thermo Fisher Scientific, 4391128). A maximum of 350 ng total RNA was incubated at 16°C for 30 min and then at 42°C for 30 min for the reverse transcription reaction. The reaction was concluded with a 5 min incubation at 85°C. The reverse transcription reaction product was pre-amplified with the Taqman PreAmp Master Mix and 5× primer pool (including *miR-31-5p*, *miR-206* and *miR-191-5p*). The pre-amplification reaction was performed with the following protocol: 95°C for 10 min, 55°C for 2 min, 72°C for 2 min; 12× cycle of a two-step cycling protocol with a 95°C for 15 s denaturation, 60°C for 4 min annealing/extension and enzyme inactivation at 99.9°C for 10 min. The pre-amplified product was then diluted by a 12.5× factor and used for the qPCR reaction. The qPCR reaction was performed with the TaqMan Fast Advanced Master Mix according to the manufacturer's instructions for CFX96 real-time detection system (Thermo Fisher Scientific, 4444557). Briefly, each reaction was run with the following run method: UNG activation (50°C for 2 min); polymerase activation (95°C for 20 s) and 40× cycles of a denaturation (95°C for 3 s) and annealing/extension (60°C for 30 s) two-step cycling. Normalization was performed to the U6 non-coding small nuclear RNA (snRNA) (Fiorillo et al., 2015) and *miR-191-5p* for RNA extracted from gastrocnemius muscle and serum RNA-extracted samples, respectively. The following miRNA assays were used: *mmu-miR-31-5p* Assay ID 000185; *U6* snRNA Assay ID 001973; *has-miR-206* Assay ID 000510; *hsa-miR-31-5p* Assay ID 002279; and *hsa-miR-191-5p* Assay ID 002299.

### Reverse transcription and qPCR reactions for *DUX4-fl* mRNA

Quantification of *DUX4-fl* transcription was performed with a nested qRT-PCR as previously described (Jones et al., 2017). Primers used for ten-cycle pre-amplification were *DUX4-fl* For, 5′-GCTCTGCTGGAGGAGCTTTAGGA-3′ and *DUX4-fl* Rev1, 5′-CGCACTGCTCGCAGGTCTGCWGGT-3′. Primers used for qPCR were *DUX4-fl* For and *DUX4-fl* Rev2, 5′-GCAGGTCTGCWGGTACCTGG-3′. Expression of *DUX4-fl* was normalized to 18S rRNA using 10 ng cDNA.

### H&E staining

Gastrocnemius muscle was dissected and then freshly frozen in Tissue-Tek OCT compound (Sakura Finetek, 4583) in cold isopentane. Briefly, 12 μm cryosections were fixed in cold acetone at −20°C for 5 min and stained with Hematoxylin for 3 min. The cryosections were subsequently incubated with differentiation solution for 20 s and Scott's Bluing reagent for 1 min. The stained cryosections were rinsed three times in a 1:2 Eosin to 70% ethanol solution, and dehydrated with a series of 30 s ethanol washes (70%, 95%) and 1 min 100% ethanol wash. The stained cryosections were cleared with xylene for 5 min and mounted. A series of three non-consecutive sections were imaged on a Leica DM2000 and analyzed with LAS 4.12 software (Leica Microsystems).

### Hydroxyproline quantification

The hydroxyproline assay was performed on gastrocnemius muscle, as previously described (Heydemann et al., 2005). Briefly, the tissue was minced overnight in 2 ml of 6 M hydrochloric acid at 110°C and the resulting hydrolysate (10 µl) was mixed with 150 µl isopropanol. Hydroxyproline oxidation was then performed for 10 min at room temperature with the addition of 72 µl of 1.4% cholramin-T in citrate buffer. Ehrlich's reagent (1 ml) was then added for 30 min at 55°C. The extinction measurement was performed at 558 nm.

### Immunofluorescence

Immunofluorescence was performed on 10 μm cryosections from OCT-embedded frozen gastrocnemius muscle as described (Jones et al., 2020). Cryosections were fixed with 4% paraformaldehyde/PBS on ice for 10 min and permeabilized with 0.25% Triton X-100/PBS for 10 min. When using mouse primary antibodies, the sections were incubated with Mouse on Mouse Detection Kit (Vector Laboratories; BMK-2202) IgG blocking solution for 1 h. Sections were subsequently incubated with a 5% normal goat serum, 2% bovine serum albumin, 0.01% TritonX-100/PBS blocking solution for 30 min and with primary antibodies at 4°C overnight. The cryosections were then incubated with secondary antibody at room temperature for 45 min on the following day and then mounted in ProLong Gold with 4′,6-diamidino-2-phenylindole (DAPI) nuclear staining. A series of three non-consecutive sections were acquired using a Leica DMi8, DFC365 FX camera and LAS X Expert software (Leica Microsystems) and used for quantification in plantaris, and medial and lateral gastrocnemius muscles. Quantifications were performed in the entire plantaris muscle, whereas quantifications in gastrocnemius muscle were performed in 1183.11 µm square crops (one crop for each gastrocnemius muscle). Images were analyzed with ImageJ 1.52i software. Primary antibodies used were as follows: anti-DUX4 (clone E5-5, Abcam, ab124699), diluted 1:200; anti-dystrophin rabbit polyclonal antibody (Abcam, ab15277), diluted 1:200; and anti-embryonic fast myosin heavy chain mouse monoclonal antibody (Developmental Studies Hybridoma Bank, F1.652), diluted 1:20. The F1.652 monoclonal antibody, developed by the Baxter Laboratory for Stem Cell Biology, Stanford University, was obtained from the Developmental Studies Hybridoma Bank, created by the Eunice Kennedy Shriver National Institute of Child Health and Human Development of the National Institutes of Health and maintained at The University of Iowa (Iowa City, IA, USA).

### *Ex vivo* muscle physiology

Specific muscle twitch, tetanus and force-frequency *ex vivo* physiology measurements were performed and analyzed in the EDL as previously described (Jones et al., 2020).

### Statistical analysis

All miScript two-group statistical analyses for miRNA profiles were performed with unpaired Student's *t*-test using the Qiagen data analysis Excel spreadsheet and according to Qiagen handbook instructions. The remaining statistical analysis was performed using GraphPad Prism 8 and described for each set of data. *n*=7-8 animals with an even number of males and females per group was used for analysis. *P*<0.05 was considered statistically significant.

## Supplementary Material

Supplementary information
