# Supplementary material for: Identification of candidate miRNA biomarkers for facioscapulohumeral muscular dystrophy using DUX4-based mouse models
Source: Dis Model Mech. 2021 Aug 24;14(8):dmm049016. doi: 10.1242/dmm.049016 (PMC8405850; doi:10.1242/dmm.049016)
Supplement: Supplementary information [file dmm-14-049016-s1.pdf]

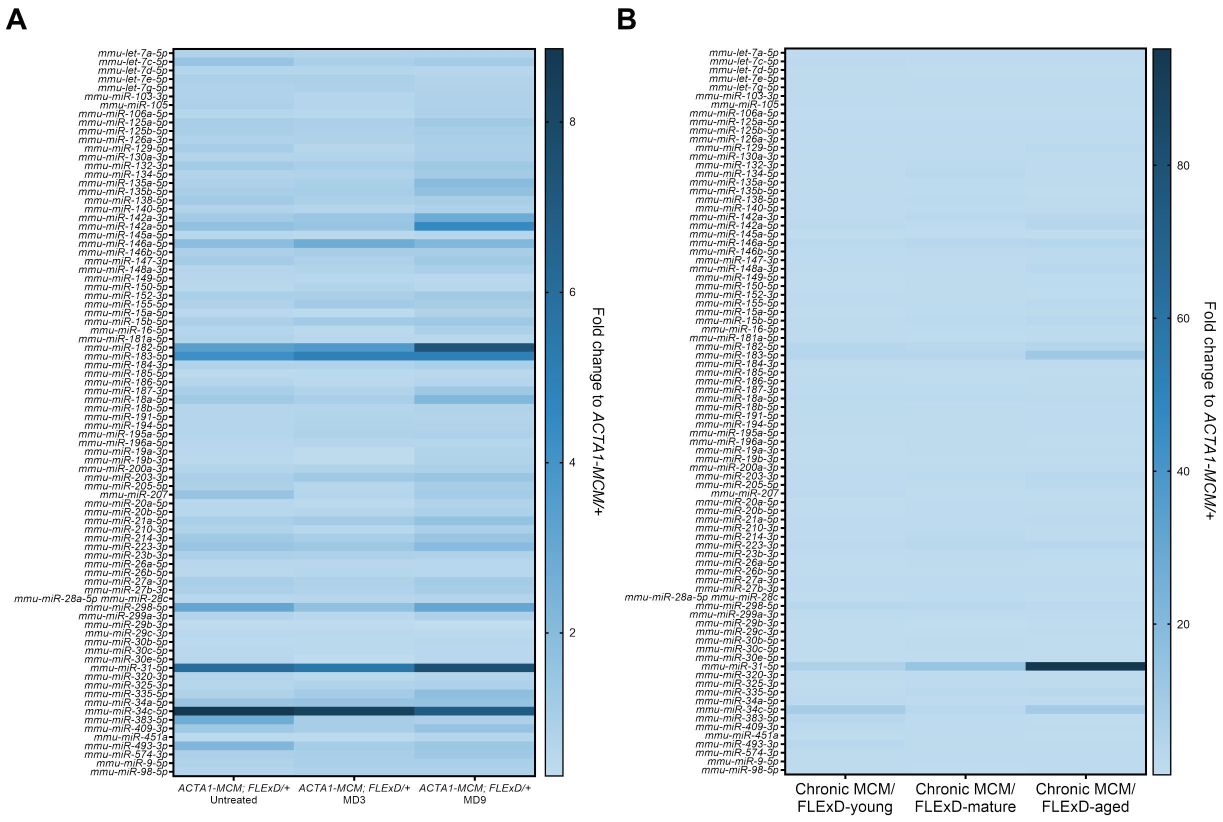


**Fig. S1. Immunopathology-associated miRNA profile in the gastrocnemius muscle from FSHD-like mouse models. (A, B)** Heat maps depicting the miRNA profile as determined using the immunopathology panel in (A) the moderate DUX4 induced FSHD-like mouse model and (B) the DUX4 chronic FSHD-like mouse models (n=8 animals for *ACTA1-MCM;FLExD/+* untreated, n=8 animals for *ACTA1-MCM;FLExD/+* MD3, n=8 animals for *ACTA1-MCM;FLExD/+* MD9, n=7 animals for *ACTA1-MCM/+* 6 months-old, n=9 animals for chronic MCM/FLExD-mature, n=6 animals for *ACTA1-MCM/+* 14-18 months-old and n=8 animals for chronic MCM/FLExD-aged experimental groups).

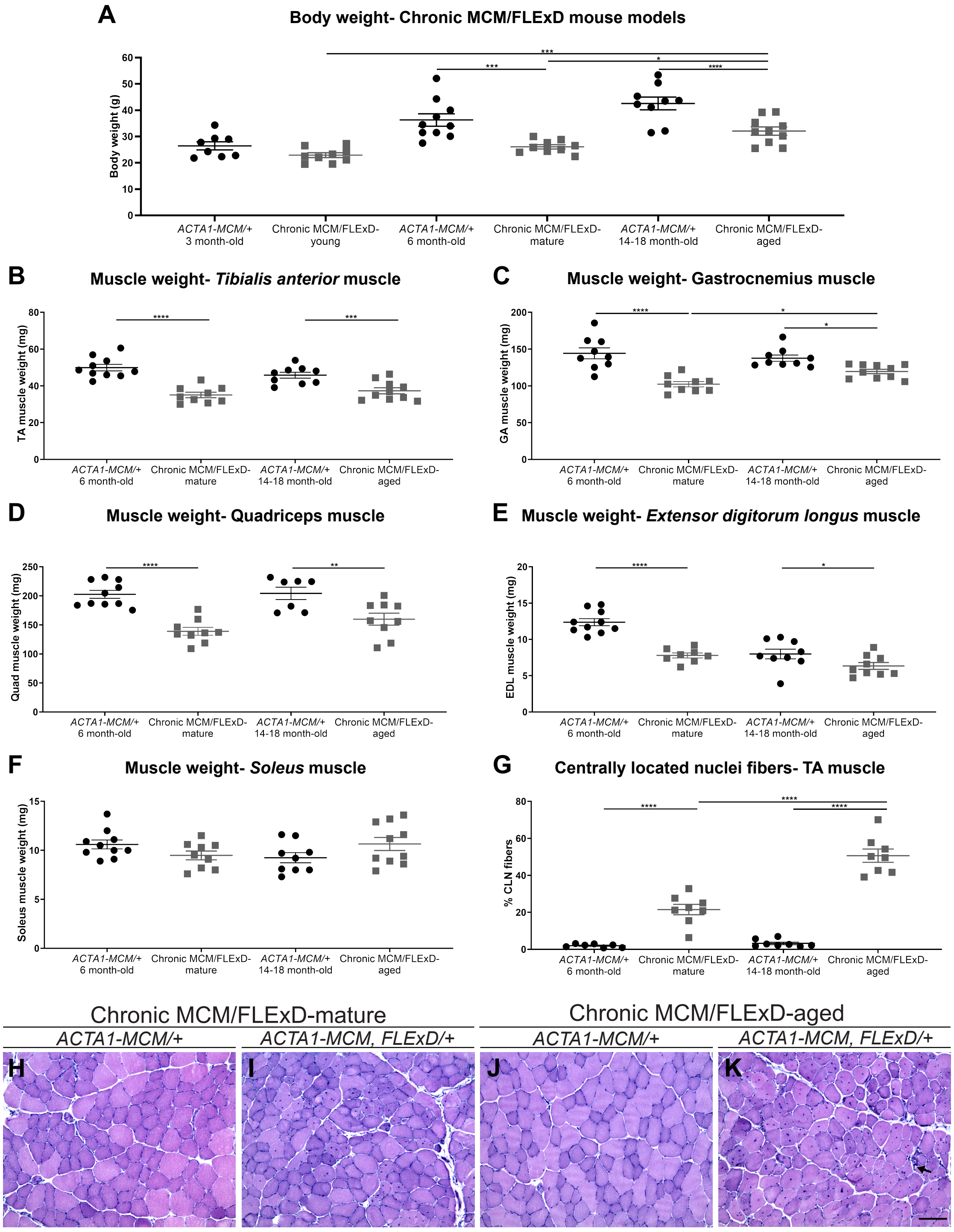


Disease Models & Mechanisms • Supplementary information

**Fig. S2. Body and muscle weight of skeletal muscles and histopathology analysis of *tibialis anterior* muscle. (A)** Body weight of chronic MCM/FLExD mouse models. Statistical analysis was performed with two tailed one-way ANOVA (uncorrected Fisher's LSD) with an n=8 animals for *ACTA1-MCM/+* 1 month-old, n=9 animals for *ACTA1-MCM;FLExD/+* 1 month-old, n=10 animals for *ACTA1-MCM/+* 6 months-old, n=9 animals for chronic MCM/FLExD-mature, n=9 animals for *ACTA1-MCM/+* 14-18 months-old and n=10 animals for chronic MCM/FLExD-aged experimental groups. **(B-F)** Muscle weight of *tibialis anterior* (n=10 animals for *ACTA1-MCM/+* 6 months-old, n=9 animals for chronic MCM/FLExD-mature, n=9 animals for *ACTA1-MCM/+* 14-18 months-old and n=10 animals for chronic MCM/FLExD-aged experimental groups) (B), gastrocnemius (n=9 animals for *ACTA1-MCM/+* 6 months-old, n=9 animals for chronic MCM/FLExD-mature, n=9 animals for *ACTA1-MCM/+* 14-18 months-old and n=10 animals for chronic MCM/FLExD-aged experimental groups) (C), quadriceps (n=9 animals for *ACTA1-MCM/+* 6 months-old, n=9 animals for chronic MCM/FLExD-mature, n=7 animals for *ACTA1-MCM/+* 14-18 months-old and n=9 animals for chronic MCM/FLExD-aged experimental groups) (D), *extensor digitorum longus* (n=10 animals for *ACTA1-MCM/+* 6 months-old, n=8 animals for chronic MCM/FLExD-mature, n=9 animals for *ACTA1-MCM/+* 14-18 months-old and n=9 animals for chronic MCM/FLExD-aged experimental groups) (E) and *soleus* (n=10 animals for *ACTA1-MCM/+* 6 months-old, n=9 animals for chronic MCM/FLExD-mature, n=9 animals for *ACTA1-MCM/+* 14-18 months-old and n=10 animals for chronic MCM/FLExD-aged experimental groups) (F) muscles. Statistical analysis was performed with two tailed one-way ANOVA (uncorrected Fisher's LSD). **(G)** Centrally located nuclei fibers quantification in *tibialis anterior* muscle. Statistical analysis was performed with two tailed one-way ANOVA (uncorrected Fisher's LSD) with an n=7 animals for *ACTA1-MCM/+* 6 months-old, n=8 animals for chronic MCM/FLExD-mature, n=8 animals for *ACTA1-MCM/+* 14-18 months-old and n=8 animals for chronic MCM/FLExD-aged experimental groups. Data is presented as mean ± s.e.m.; * $p<0.05$, ** $p<0.01$, ***$p<0.001$, ****$p<0.0001$. **(H-K)** Hematoxylin and Eosin staining of *tibialis anterior* (TA) muscles from (H, I) chronic MCM/FLExD-mature mice and age matched 6 month-old *ACTA1-MCM/+* controls and (J, K) chronic MCM/FLExD-mature mice and age matched 14-18 months-old *ACTA1-MCM/+* controls. Black arrow (K) depicts a damaged fiber with immune infiltrate. Representative images from n=3 animals for each experimental group Bar = 50 μm.

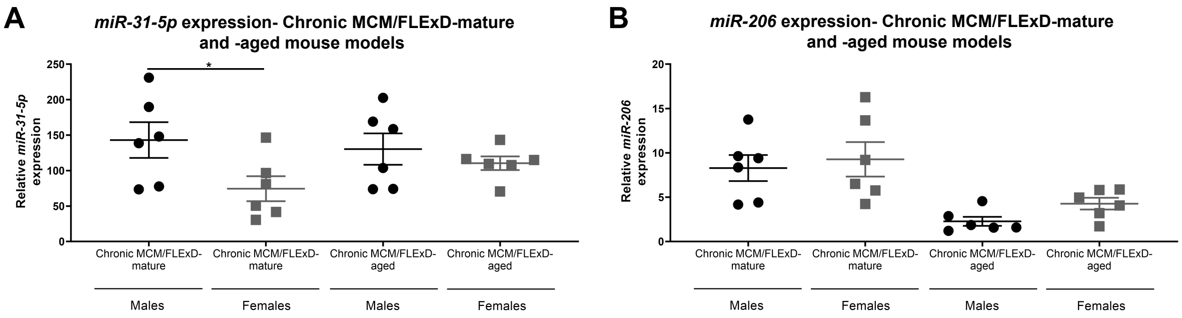


**Fig. S3. miRNA expression in the chronic DUX4 mouse models.** **(A)** *miR-31-5p* expression in the gastrocnemius muscle from male and female chronic MCM/FLExD-mature and –aged mouse models. **(B)** *miR-206* expression in the gastrocnemius muscle from male and female chronic MCM/FLExD-mature and –aged mouse models. Statistical analysis was performed with two-tailed one-way ANOVA (uncorrected Fisher's LSD) with an n=6 animals for chronic MCM/ FLExD-mature and aged experimental groups. Data is presented as mean ± s.e.m.; * $p<0.05$.

Disease Models & Mechanisms • Supplementary information

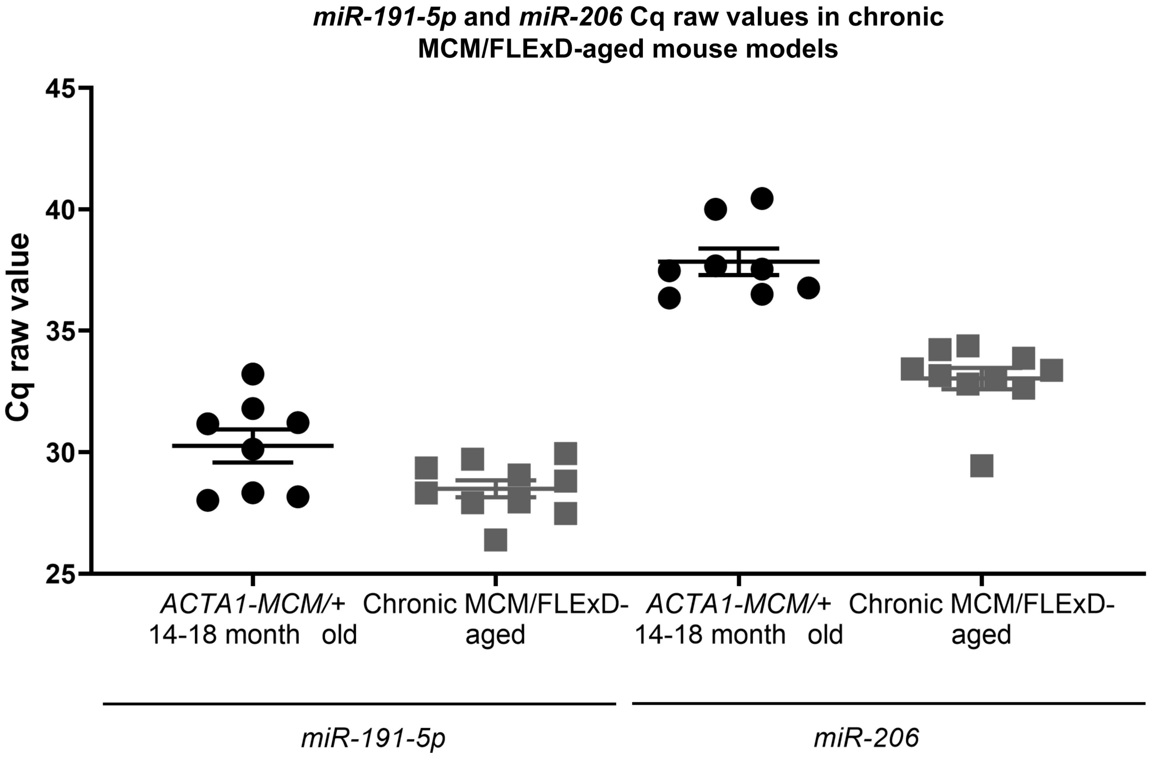


**Fig. S4. Serum analysis of *mi-191-5p* and *miR-206* expression in the chronic MCM/FLExD-aged mouse model.** Cq raw values for *miR-191-5p* and *miR-206* expression in the serum from *ACTA1-MCM/+* 14-18-month-old and chronic MCM/FLExD-aged mice.

Disease Models & Mechanisms • Supplementary information

**Table S1. Fold change relative expression raw data for miScript analysis.** Raw fold change values for relative expression in the miRNA profile panel. Red boxes: Statistically significant upregulation, fold change above a 2-fold threshold. Blue boxes: Statistically significant downregulation, fold change below 0.5-fold change. Gray boxes: Statistically significant fold changes above or below the defined analysis threshold.

| miRNA ID | MD3 | MD9 | 1 month old | 6 months old | 14-18 months old |
|---|---|---|---|---|---|
| *mmu-let-7a-5p* | 0.73 | 0.76 | 0.81 | 0.85 | 1.01 |
| *mmu-let-7c-5p* | 0.96 | 1.12 | 1.52 | 1.02 | 1.04 |
| *mmu-let-7d-5p* | 0.67 | 0.60 | 0.66 | 0.55 | 0.58 |
| *mmu-let-7e-5p* | 0.89 | 0.73 | 0.90 | 1.10 | 0.90 |
| *mmu-let-7g-5p* | 0.85 | 0.75 | 0.74 | 0.84 | 0.72 |
| *mmu-miR-103-3p* | 0.65 | 0.82 | 0.77 | 0.92 | 0.93 |
| *mmu-miR-105* | 0.67 | 0.81 | 0.85 | 0.84 | 0.83 |
| *mmu-miR-106a-5p* | 0.56 | 0.85 | 0.61 | 0.55 | 0.50 |
| *mmu-miR-125a-5p* | 0.93 | 1.27 | 1.00 | 0.93 | 0.96 |
| *mmu-miR-125b-5p* | 0.87 | 1.02 | 0.98 | 1.19 | 0.97 |
| *mmu-miR-126a-3p* | 0.79 | 0.95 | 0.78 | 0.76 | 0.62 |
| *mmu-miR-129-5p* | 0.61 | 0.93 | 1.05 | 0.94 | 1.78 |
| *mmu-miR-130a-3p* | 0.71 | 0.94 | 0.69 | 0.99 | 0.78 |
| *mmu-miR-132-3p* | 0.99 | 1.32 | 1.18 | 1.51 | 1.15 |
| *mmu-miR-134-5p* | 0.74 | 1.17 | 0.72 | 2.52 | 1.00 |
| *mmu-miR-135a-5p* | 0.96 | 1.99 | 0.84 | 1.15 | 0.75 |
| *mmu-miR-135b-5p* | 1.02 | 1.67 | 0.98 | 0.96 | 0.40 |
| *mmu-miR-138-5p* | 0.93 | 0.98 | 1.18 | 1.35 | 0.67 |
| *mmu-miR-140-5p* | 0.65 | 0.86 | 0.76 | 0.90 | 0.76 |
| *mmu-miR-142a-3p* | 1.40 | 2.80 | 1.21 | 2.02 | 2.84 |
| *mmu-miR-142a-5p* | 1.53 | 4.50 | 1.69 | 0.87 | 3.46 |
| *mmu-miR-145a-5p* | 0.45 | 0.59 | 0.56 | 0.58 | 0.42 |
| *mmu-miR-146a-5p* | 2.74 | 2.19 | 1.85 | 2.75 | 3.31 |
| *mmu-miR-146b-5p* | 1.01 | 1.03 | 0.91 | 1.47 | 1.54 |
| *mmu-miR-147-3p* | 0.85 | 1.26 | 1.10 | 0.95 | 1.37 |
| *mmu-miR-148a-3p* | 0.72 | 0.95 | 0.67 | 1.17 | 2.14 |
| *mmu-miR-149-5p* | 0.54 | 0.49 | 0.60 | 0.84 | 0.74 |
| *mmu-miR-150-5p* | 0.71 | 0.56 | 0.51 | 0.74 | 0.97 |
| *mmu-miR-152-3p* | 0.97 | 1.19 | 0.96 | 0.83 | 0.59 |
| *mmu-miR-155-5p* | 1.17 | 1.07 | 0.82 | 1.17 | 1.70 |
| *mmu-miR-15a-5p* | 0.41 | 0.82 | 0.48 | 0.92 | 0.70 |
| *mmu-miR-15b-5p* | 1.15 | 1.32 | 0.91 | 1.35 | 2.01 |
| *mmu-miR-16-5p* | 0.44 | 0.89 | 0.65 | 1.05 | 0.83 |
| *mmu-miR-181a-5p* | 0.53 | 0.53 | 0.71 | 0.47 | 0.71 |
| *mmu-miR-182-5p* | 3.68 | 7.36 | 3.42 | 2.03 | 3.82 |
| *mmu-miR-183-5p* | 4.98 | 4.97 | 4.19 | 3.87 | 11.10 |
| *mmu-miR-184-3p* | 0.78 | 0.96 | 0.75 | 1.00 | 0.98 |
| *mmu-miR-185-5p* | 0.46 | 0.48 | 0.43 | 0.35 | 0.31 |
| *mmu-miR-186-5p* | 0.50 | 0.67 | 0.65 | 0.59 | 0.72 |
| *mmu-miR-187-3p* | 0.76 | 1.31 | 1.00 | 1.03 | 0.65 |
| *mmu-miR-18a-5p* | 1.00 | 2.18 | 1.28 | 1.36 | 1.42 |
| *mmu-miR-18b-5p* | 0.67 | 0.77 | 0.64 | 0.84 | 0.86 |
| *mmu-miR-191-5p* | 0.72 | 0.70 | 0.65 | 0.73 | 0.91 |
| *mmu-miR-194-5p* | 0.74 | 0.74 | 0.67 | 0.80 | 1.04 |
| *mmu-miR-195a-5p* | 0.80 | 0.86 | 0.67 | 0.95 | 0.89 |
| *mmu-miR-196a-5p* | 0.63 | 0.61 | 0.55 | 0.67 | 0.55 |
| *mmu-miR-19a-3p* | 0.36 | 0.71 | 0.48 | 0.72 | 0.70 |
| *mmu-miR-19b-3p* | 0.37 | 0.76 | 0.46 | 0.49 | 0.42 |
| *mmu-miR-200a-3p* | 0.76 | 0.90 | 0.68 | 0.84 | 1.00 |
| *mmu-miR-203-3p* | 1.27 | 1.47 | 0.97 | 1.34 | 2.31 |
| *mmu-miR-205-5p* | 0.61 | 1.05 | 0.92 | 0.66 | 1.62 |
| *mmu-miR-207* | 0.67 | 1.10 | 1.49 | 0.58 | 0.44 |
| *mmu-miR-20a-5p* | 0.46 | 0.76 | 0.55 | 1.00 | 0.73 |
| *mmu-miR-20b-5p* | 0.63 | 0.87 | 0.56 | 0.80 | 0.68 |
| *mmu-miR-21a-5p* | 1.17 | 1.61 | 0.96 | 1.15 | 1.42 |
| *mmu-miR-210-3p* | 0.57 | 0.96 | 0.75 | 0.80 | 0.83 |
| *mmu-miR-214-3p* | 1.00 | 1.48 | 1.25 | 1.88 | 1.05 |
| *mmu-miR-223-3p* | 1.36 | 1.99 | 1.44 | 2.41 | 2.92 |
| *mmu-miR-23b-3p* | 0.86 | 0.80 | 0.91 | 0.97 | 0.88 |
| *mmu-miR-26a-5p* | 0.53 | 0.57 | 0.53 | 0.74 | 0.60 |
| *mmu-miR-26b-5p* | 0.60 | 0.57 | 0.51 | 0.61 | 0.60 |
| *mmu-miR-27a-3p* | 0.81 | 1.18 | 0.99 | 1.09 | 1.14 |
| *mmu-miR-27b-3p* | 0.78 | 1.03 | 0.90 | 1.05 | 1.10 |
| *mmu-miR-28a-5p mmu-miR-28c* | 0.58 | 0.69 | 0.57 | 0.98 | 0.53 |
| *mmu-miR-298-5p* | 1.72 | 3.04 | 3.03 | 2.08 | 1.57 |
| *mmu-miR-299a-3p* | 0.48 | 0.85 | 0.70 | 0.90 | 1.07 |
| *mmu-miR-29b-3p* | 0.39 | 0.32 | 0.36 | 0.28 | 0.35 |
| *mmu-miR-29c-3p* | 0.52 | 0.47 | 0.49 | 0.57 | 0.67 |
| *mmu-miR-30b-5p* | 0.46 | 0.53 | 0.42 | 0.64 | 0.60 |
| *mmu-miR-30c-5p* | 0.47 | 0.52 | 0.50 | 0.66 | 0.59 |
| *mmu-miR-30e-5p* | 0.33 | 0.55 | 0.54 | 0.60 | 0.52 |
| *mmu-miR-31-5p* | 5.51 | 7.56 | 6.06 | 13.78 | 95.20 |
| *mmu-miR-320-3p* | 0.48 | 0.70 | 0.68 | 0.87 | 0.63 |
| *mmu-miR-325-3p* | 0.78 | 0.86 | 0.61 | 0.85 | 0.79 |
| *mmu-miR-335-5p* | 1.13 | 1.84 | 0.79 | 1.61 | 2.42 |
| *mmu-miR-34a-5p* | 1.54 | 1.62 | 1.44 | 1.23 | 0.94 |
| *mmu-miR-34c-5p* | 8.31 | 6.97 | 8.86 | 1.98 | 9.73 |
| *mmu-miR-383-5p* | 1.05 | 0.84 | 2.69 | 1.19 | 0.58 |
| *mmu-miR-409-3p* | 1.01 | 1.55 | 1.30 | 1.12 | 0.69 |
| *mmu-miR-451a* | 0.41 | 0.67 | 0.47 | 0.72 | 0.35 |
| *mmu-miR-493-3p* | 1.08 | 1.38 | 2.21 | 0.75 | 0.73 |
| *mmu-miR-574-3p* | 0.99 | 1.29 | 0.92 | 1.14 | 0.92 |
| *mmu-miR-9-5p* | 0.95 | 0.95 | 0.75 | 1.22 | 1.05 |
| *mmu-miR-98-5p* | 0.81 | 0.82 | 0.68 | 0.53 | 0.70 |

**Table S2. Participant information.** DRA = D4Z4 reduced allele. NA = Not applicable. Shortest chromosome 4qA D4Z4 EcoRI/BlnI fragment is >41kb and therefore not FSHD1.

| Subject ID | Gender | Age | Status | DRA (EcoRI/BlnI) |
|---|---|---|---|---|
| 1 | Male | 78 | Clinically affected FSHD1 | 19kb |
| 2 | Female | 31 | Clinically affected FSHD1 | 18kb |
| 3 | Male | 60 | Clinically affected FSHD1 | 22kb |
| 4 | Male | 53 | Clinically affected FSHD1 | 26kb |
| 5 | Male | 79 | Clinically affected FSHD1 | 34kb |
| 6 | Male | 40 | Clinically affected FSHD1 | 22kb |
| 7 | Male | 48 | Clinically affected FSHD1 | 22kb |
| 8 | Female | 51 | Clinically affected FSHD1 | 16kb |
| 9 | Male | 17 | Clinically affected FSHD1 | 16kb |
| 10 | Male | 32 | Clinically affected FSHD1 | 24kb |
| 11 | Male | 56 | Clinically affected FSHD1 | 26kb |
| 12 | Female | 72 | Clinically affected FSHD1 | 27kb |
| 13 | Female | 72 | Clinically affected FSHD1 | 27kb |
| 14 | Male | 30 | Clinically affected FSHD1 | 27kb |
| 15 | Male | 59 | Clinically affected FSHD2 | 42kb |
| 16 | Male | 51 | Healthy | NA |
| 17 | Male | 37 | Healthy | NA |
| 18 | Female | 68 | Healthy | NA |
| 19 | Female | 52 | Healthy | NA |
| 20 | Female | 57 | Healthy | NA |
| 21 | Male | 43 | Healthy | NA |
| 22 | Male | 66 | Healthy | NA |
| 23 | Male | 81 | Healthy | NA |
| 24 | Male | 47 | Healthy | NA |
| 25 | Male | 52 | Healthy | NA |
| 26 | Female | 36 | Healthy | NA |
| 27 | Female | 32 | Healthy | NA |
| 28 | Female | 24 | Healthy | NA |
| 29 | Male | 77 | Healthy | NA |
| 30 | Male | 73 | Healthy | NA |
